# Characterization of Chronic Lymphocytic Leukemia Immunoglobulin Rearrangements from Partial Read Sequencing

**DOI:** 10.1093/gpbjnl/qzaf041

**Published:** 2025-05-02

**Authors:** Azahara Fuentes-Trillo, Alicia Serrano-Alcalá, Blanca Ferrer-Lores, Laura Ventura-López, Enrique Seda, Ana-Bárbara García-García, Blanca Navarro, María José Terol, Felipe Javier Chaves

**Affiliations:** Genomic and Diabetes Unit, INCLIVA Biomedical Research Institute, Valencia 46010, Spain; Hematology Service, Valencia University Clinical Hospital, Valencia 46010, Spain; Physiology Department, University of Valencia, Valencia 46010, Spain; Lymphoproliferative Syndrome Group, INCLIVA Biomedical Research Institute, Valencia 46010, Spain; Hematology Service, Valencia University Clinical Hospital, Valencia 46010, Spain; Lymphoproliferative Syndrome Group, INCLIVA Biomedical Research Institute, Valencia 46010, Spain; Hematology Service, Valencia University Clinical Hospital, Valencia 46010, Spain; Lymphoproliferative Syndrome Group, INCLIVA Biomedical Research Institute, Valencia 46010, Spain; Genomic and Diabetes Unit, INCLIVA Biomedical Research Institute, Valencia 46010, Spain; Genomic and Diabetes Unit, INCLIVA Biomedical Research Institute, Valencia 46010, Spain; CIBERDEM, ISCIII, Madrid 28029, Spain; Hematology Service, Valencia University Clinical Hospital, Valencia 46010, Spain; INCLIVA Biomedical Research Institute, Valencia 46010, Spain; Hematology Service, Valencia University Clinical Hospital, Valencia 46010, Spain; Lymphoproliferative Syndrome Group, INCLIVA Biomedical Research Institute, Valencia 46010, Spain; Medicine Department, University of Valencia, Valencia 46010, Spain; Genomic and Diabetes Unit, INCLIVA Biomedical Research Institute, Valencia 46010, Spain; CIBERDEM, ISCIII, Madrid 28029, Spain

**Keywords:** Chronic lymphocytic leukemia, *IGH* locus, NGS, B cell, Immune repertoire

## Abstract

The determination of the mutational status in the immunoglobulin variable region is an established prognostic biomarker for chronic lymphocytic leukemia (CLL). The length and inner variability of the variable, diversity, and joining (VDJ) rearranged sequences compromise B-cell clone characterization using next-generation sequencing (NGS), and a standardization is needed to adapt the procedure to the current clinical guidelines. Here, we develop a complete strategy for sequencing the variable domain of the immunoglobulin heavy chain (*IGH*) locus with a simple, low-cost, and efficient method that enables sequencing using shorter reads (MiSeq 150 × 2), allowing for faster results. Clonality and mutational status determination are performed within the same analysis pipeline. We tested and validated the method using 319 CLL patients previously diagnosed with *IGH* locus characterized using Sanger sequencing, along with 47 healthy donor samples. The analysis method follows a clone-centered consensus sequence strategy to identify B-cell clones and establish a clonal threshold specific for each patient’s clonality profile, thereby overcoming the limitations of Sanger sequencing which is the gold standard used for determining immunoglobulin heavy variable (*IGHV*) mutational status.

## Graphical abstract

**Graphical Abstract:**
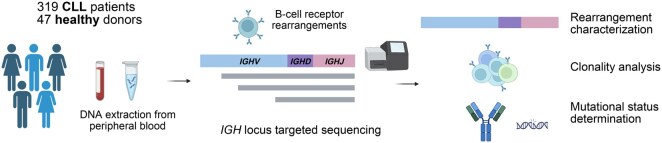

## Introduction

Chronic lymphocytic leukemia (CLL) is characterized by the proliferation of mature malignant B lymphocytes expressing CD5, CD19, and CD23 and low expression of CD20 and CD79b, with variable lymphocyte stages. Despite this phenotypic homogeneity, CLL presents a highly heterogeneous course and significant genomic changes, and this biological diversity, in part, can be attributed to the immunogenetic origin of the disease [1]. Somatic hypermutation (SHM) status of the rearranged immunoglobulin heavy variable (*IGHV*) gene in B-cell receptor (BCR) is clinically relevant as a key to accurate risk stratification in CLL: patients with no or limited SHM (unmutated, UM) usually experience an aggressive course; while those with a significant SHM load (mutated, MM) follow a more indolent disease [2–4]. Unlike other alterations, this biomarker is stable over time and should be assessed prior to treatment in all patients with CLL [5].

Current standard protocols support the use of Sanger sequencing (SSeq) to characterize the mutational status in the tumor clone [6]. However, a non-negligible number of cases with single unproductive or multiple productive tumor clonotypes (3%–4%), remain unclear when applying Sanger-based methodologies and due to the lack of characterizing the clonal abundance [6–8]. In this regard, next-generation sequencing (NGS) allows a far more detailed view of the BCR immunoglobulin (IG) repertoires and provides a deeper insight into the biology of CLL [9,10]. Several studies [9,11,12] have reported NGS-based analysis which can reveal the existence of multiple productive rearrangements in up to 25% of patients tested, with minor related subclones arising due to intraclonal diversification [10] or unrelated clonotypes [13]. These facts highlight the need to clarify amplification biases and quantification issues as well as the paucity of multicenter-validated protocols to establish a consensus clonal threshold to facilitate the standardization of immunogenetic analysis in CLL with the application of novel NGS methodologies in clinical practice [14].

Immunoglobulin heavy chain (*IGH*) locus recombination in B cells involves the random selection of one fragment of each of the gene segments termed variable, diversity, and joining (VDJ) [15]. During the process, junctional diversity is generated by the insertion and deletion of random nucleotides at the recombination spots, generating a highly variable region named complementary determining region 3 (CDR3), which is folded into a protein loop key for antigen recognition. Allelic exclusion ensures that recombination of the second *IGH* locus allele copy is silenced if the first allele results in a functional rearrangement [16].

When the receptor protein is expressed on the surface of the naïve B lymphocyte, activation by exposure to a related antigen drives additional BCR diversity generated by the SHM process for affinity maturation, which introduces mutations into the *IGHV@* region [17]. Although several methods for lymphocyte repertoire NGS analysis have been described, some aspects remain challenging [18].

RNA-based library preparation methods allow the use of sequencing kits with fewer cycles due to the lack of intronic sequences. However, DNA permits the detection of unproductive recombination products and less biased estimation of clonal distribution that can happen with RNA material due to BCR differential expression among cells [19,20]. Standard degenerate primers cover the *IGHV@* leader and framework regions (FR1, FR2, and FR3). High accuracy is required to determine *IGHV@-*specific mutations, and given the higher error or cost rates with long read sequencing platforms, 2 × 300 bp sequencing on the MiSeq platform (Illumina, San Diego, CA) is the preferred method for genomic DNA (gDNA)-based libraries, using leader or FR1 primers [19]. However, using 2 × 150 bp sequencing improves read quality, reduces costs and time, and allows the use of higher capacity systems (NextSeq or higher). In addition, 2 × 150 bp reactions are common for CLL somatic mutation panels, and both can be combined in the same sequencing experiment [21,22].

**Figure 1:**
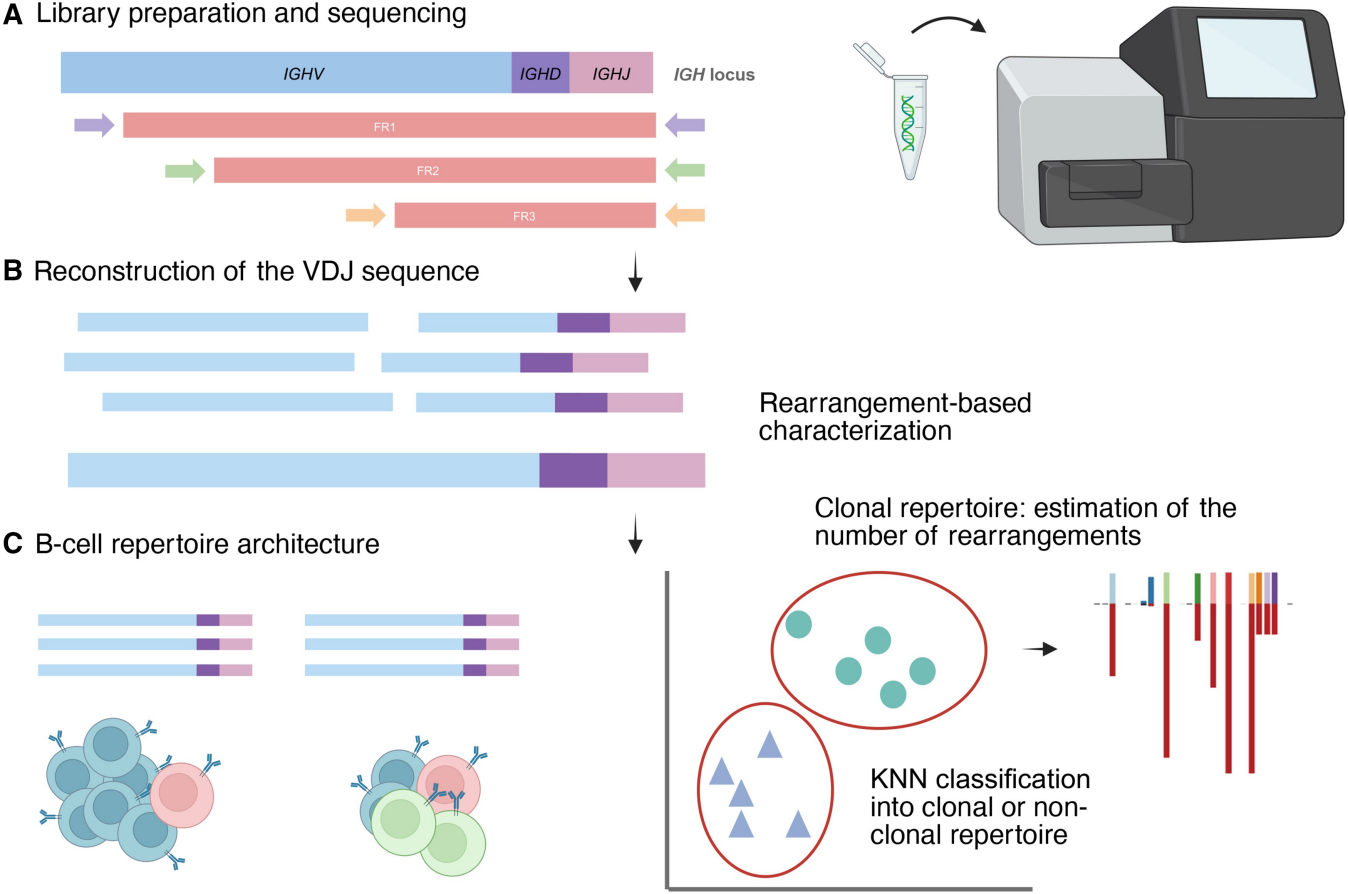
Workflow for ***IGH*** locus characterization in CLL patients **A**. Sequencing with multiplexed FR primer sets and Illumina MiSeq 2 × 150 bp kit. **B**. Reconstruction of the region of interest by overlapping FR reads using the in-house pipeline B-MyRepCLL (https://github.com/afuentri/B-MyRepCLL). The pipeline generates a consensus sequence for each B-cell rearrangement followed by filtering steps to minimize artifacts. **C**. Repertoire structure determination. Automatic KNN classification distinguishes between CLL and healthy repertoires, followed by prioritization of the predominant rearrangements to identify clonal rearrangements (clonal/subclonal cut-off). VDJ, variable, diversity, and joining; KNN, K-nearest neighbors; FR, framework region; CLL, chronic lymphocytic leukemia; IGHV, immunoglobulin heavy chain variable; IGHD, immunoglobulin heavy chain diversity; IGHJ, immunoglobulin heavy chain joining.

A reliable and automated method for sequencing IG clones in CLL patients using short reads (Illumina 2 × 150 bp), as well as the subsequent analysis to determine clonal rearrangements, CDR3 composition, and their mutational status, is presented in **Figure 1**. The VDJ region is reconstructed by integration of the three FR amplicons with a specific bioinformatics workflow for the library design. To report only clones that are determinant in clinical decisions, a K-nearest neighbors (KNN) model was employed to differentiate between healthy and CLL patients, and after the classification, a cut-off between the clonal and subclonal fractions was calculated.

## Results

**Figure 2:**
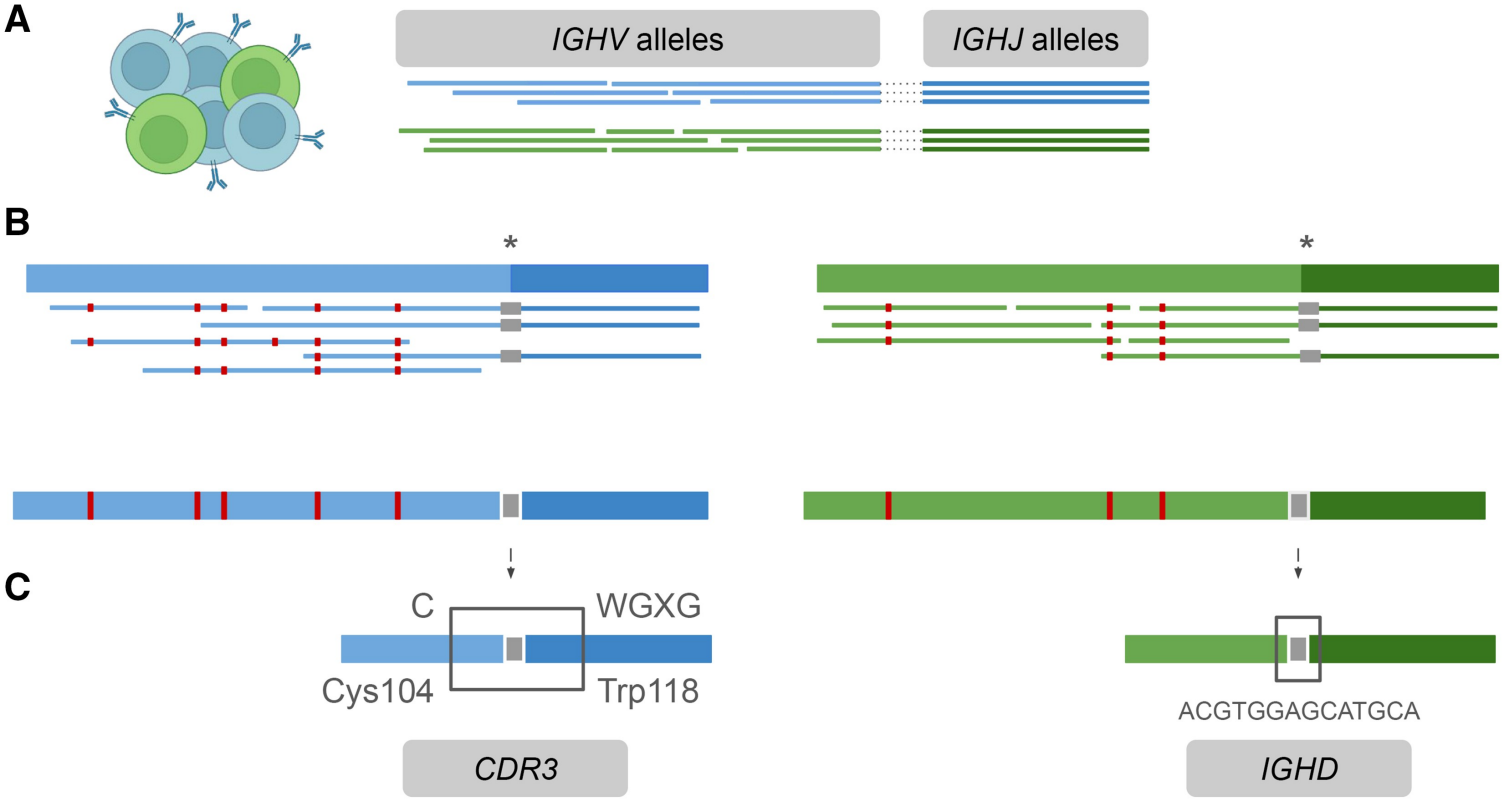
Bioinformatics pipeline basis **A**. Reads from theoretical clonal IG rearrangements in a single sample (different colors represent different B-cell clones) are initially assigned independently to different *IGHV@* and *IGHJ@* alleles. Dotted lines indicate reads aligned to both V and J alleles. **B**. In the next step, these reads are used to infer the V–J allele combinations. Reads corresponding to each *IGHV*–*IGHJ* pairing are isolated and mapped against a joined reference of the specific *IGHV*–*IGHJ* pair. A consensus sequence is generated for each combination, representing an individual IG rearrangement. These consensus sequences are used to calculate the percentage identity against germline *IGHV@* alleles. Asterisks represent the gap for *IGHD* sequence. Red and gray boxes indicate somatic hypermutation events and the junction sequence which is a gap in the reference alleles combination, respectively. **C**. *CDR3* and *IGHD* sequence extraction is performed. CDR3 amino acid sequence is retrieved by searching for the conserved amino acid motifs (Cys104, Typ118, and WGXG) in different open reading frames. *IGHD@* is detected as an insertion considering the combined sequences of *IGHV*–*IGHJ* alleles as reference. IG, immunoglobulin; CDR3, complementary determining region 3.

We developed an analysis pipeline for a short-read sequencing library design (MiSeq 2 × 150 bp), to detect clonal IG rearrangements and determine their mutational status along with the clonally expanded fraction in each patient (**Figure 2**).

The average coverage breadth above 500 reads for the IG clonal rearrangements characterized was 85% (clonal percentages ranging from 2% to 100%) (Figure S1). The percentages of the rearrangements tagged as subclonal varied from 0.1% to 9.1% of total reads assigned to *IGH* rearrangements.

### Optimization of the procedure and initial testing

**Figure 3:**
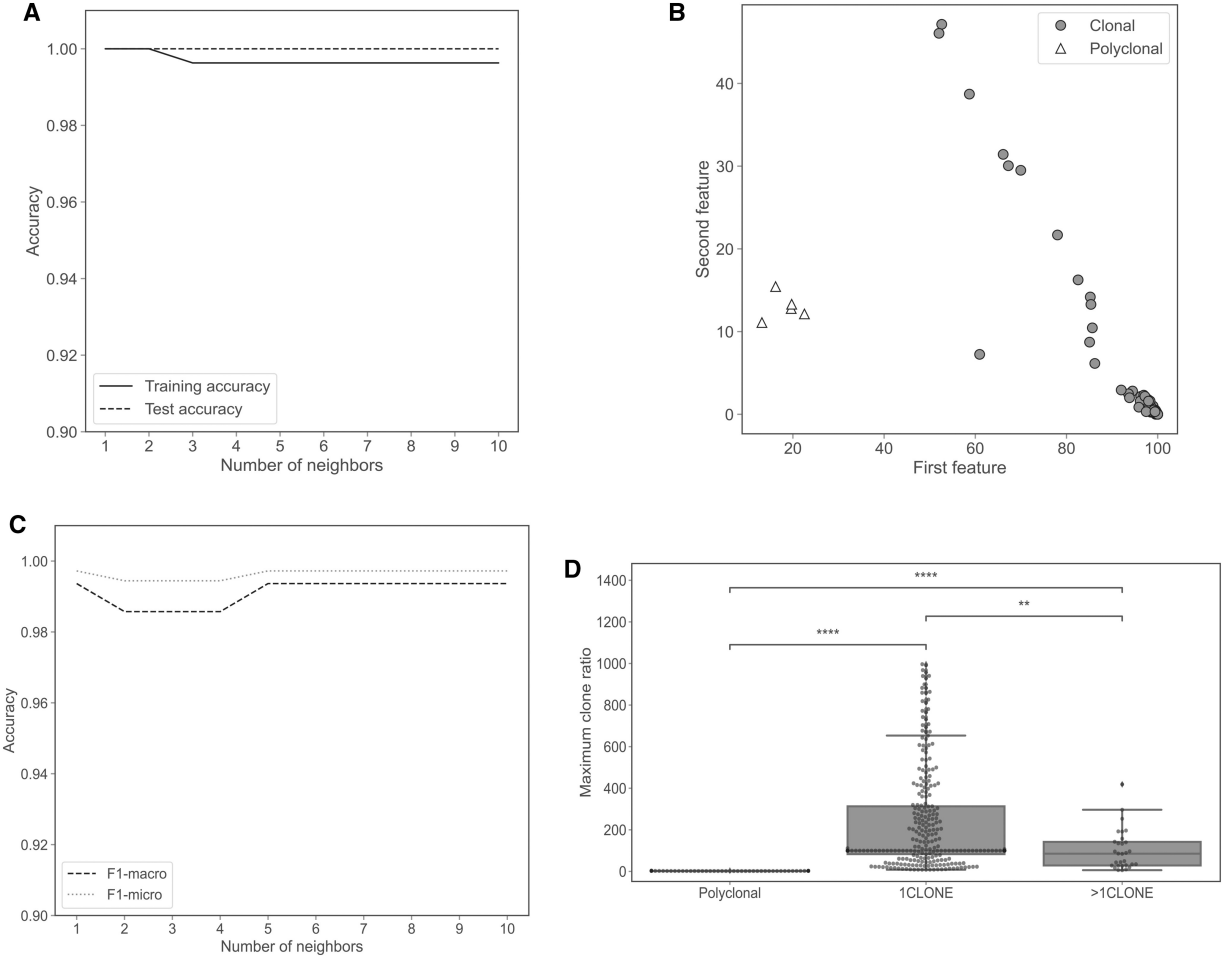
Optimization of the procedure **A**. Accuracy of KNN classification (*k* = 1–10) in a random split of the dataset between training and test sets. **B**. Scatter plot of the KNN test classification with clonal and polyclonal labels. **C**. F1-micro and F1-macro average accuracy scores for 10-fold cross-validation for KNN classification (*k* = 1–10). **D**. Box plot for MAX_DIFF values per sample grouped by polyclonal, 1CLONE, and >1CLONE. After Mann-Whitney U test, Bonferroni-corrected *P* values are annotated to show differences between group distributions (polyclonal *vs*. 1CLONE: *P* = 1.303E−27; polyclonal *vs*. >1CLONE: *P* = 5.562E−13; 1CLONE *vs.* >1CLONE: *P* = 3.213E−03). ns, not significant (*P* > 0.05); *, 0.01 < *P* ≤ 0.05; **, 0.001 < *P* ≤ 0.01; ***, 0.0001 < *P* ≤ 0.001; ****, *P* ≤ 0.0001. The scipy.stats Python module was used to perform the statistical test. MAX_DIFF maximum clonal difference within a sample.

Classification of patients into clonal and polyclonal B-cell repertoires was trained using a KNN model, to automatically detect those with expanded B-cell rearrangements. The B-cell clone data of 314 CLL patients and 47 healthy donors were subjected to random split into training (*n* = 90) and test (*n* = 271) datasets. The prediction accuracy values in the training and test datasets were 0.996 and 1.0, respectively, for *k* = 3 (**Figure 3**A and B). The mean accuracy values for 10-fold cross-validation (Figure 3C) and *k* = 3 are F1-macro = 0.99 ± 0.02 and F1-micro = 1.00 ± 0.01, respectively.

**Figure 4:**
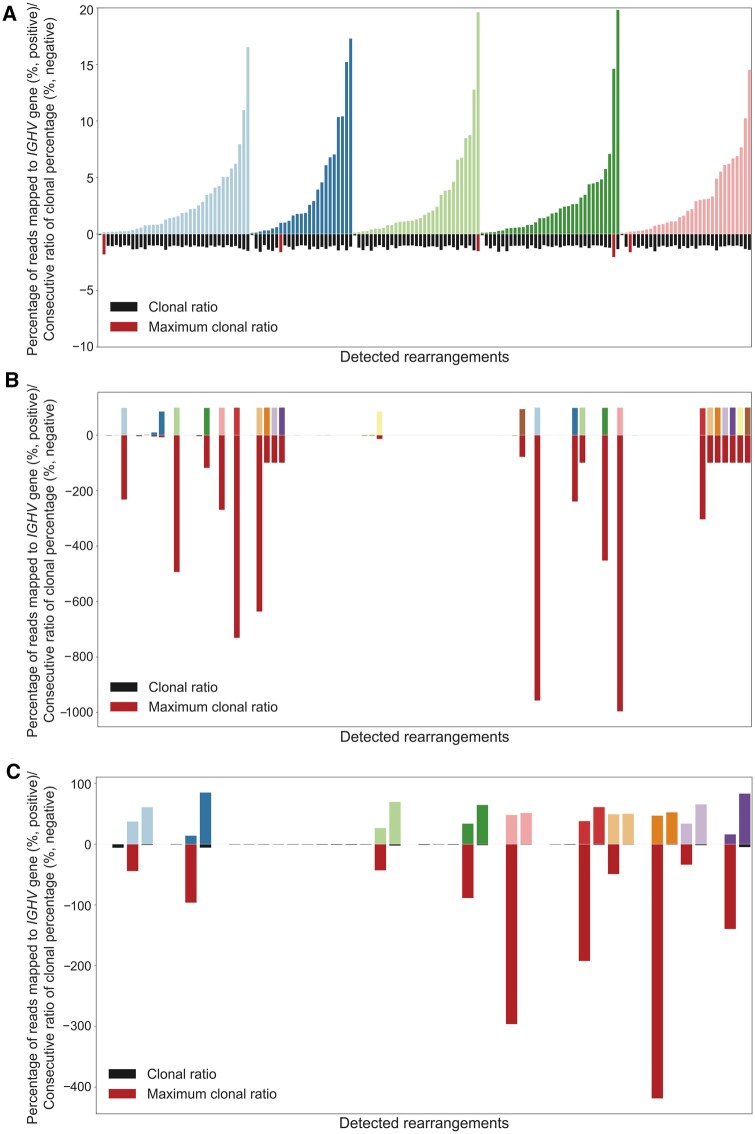
Representation of clonal ratios in polyclonal (healthy), 1CLONE, and 2CLONE groups from the test samples Bars on the positive Y-axis represent the clonal percentage of the different IG rearrangements detected per individual, with different colors per donor. Bars on the negative Y-axis represent clonal percentage ratios between consecutive clones in a sample, ordered by abundance. The maximum clonal ratio is highlighted in red and the remaining clonal ratios in black. **A**. Polyclonal repertoire (five healthy donors). **B**. 1CLONE repertoire (24 samples with a single predominant clone). **C**. 2CLONE repertoire (10 samples with double predominant rearrangements).

To determine the clonal *vs*. subclonal background in the clonal samples, difference ratios between consecutive clones encountered within a sample were calculated. **Figure 4** shows differences in the maximum clonal difference within a sample (MAX_DIFF) parameter within each of the three groups: polyclonal group (healthy donors; Figure 4A), 1CLONE group (Figure 4B), and 2CLONE group (Figure 4C). Average MAX_DIFF values were 115-fold and 50-fold higher in the 1CLONE and >1CLONE groups than the polyclonal group; *P* values after Bonferroni correction were 1.303E−27 and 5.562E−13, respectively (minimum MAX_DIFF value in the 1CLONE group was 8.19) (**Table 1**). Differences were less significant (*P* = 3.213E−03) between the 1CLONE and >1CLONE groups (Figure 3D).

**Table 1:** Average, maximum, and minimum values for MAX_DIFF in three clonal groups

Group	Average MAX_DIFF	Maximum MAX_DIFF	Minimum MAX_DIFF
1CLONE	234.79	996.52	8.19
>1CLONE	102.86	418.82	6.05
Polyclonal	2.04	3.77	1.50

*Note*: MAX_DIFF, maximum clonal difference within a sample.

The number of rearrangements considered clonal was validated in the set of 284 single-clone CLL samples and 30 CLL samples predicted as having more than 1 clonal rearrangement. Multiple-clone samples included 7 cases only detected first with NGS and confirmed with SSeq after directed SSeq with the *IGHV@* family-specific primers (Table S1). Additionally, 9 rearrangements not detected by SSeq were confirmed with GeneScan (Figures S2–S10; Table S2).

### Sensitivity and specificity

Nine additional rearrangements from both groups (single and multiple clones) in 8 samples were categorized as false positive (FP) after GeneScan validation (Figures S11–S18; Table S3). In 5 of these 9 cases, the FP rearrangement shared CDR3 with a confirmed positive rearrangement, probably owing to noise from predominant IG rearrangements. MiXCR did not report those FP clones, but some other cases (FP2 *IGHV1-3*, FP5, and FP7), where *IGHV@* genes from the FP rearrangements are reported in similar proportions (Table S3). MiXCR reports similar clonal percentages as well for the additional clones (Table S2).

In summary, the specificity for the number of rearrangements detected was 97.45% [TN/(TN + FP) = 306/314] (TN, true negative), and 100% in the case of sensitivity, as there were no false negatives (FNs).

### Clone characterization

Confirmed SSeq IG rearrangements for each of these samples were compared to those obtained by NGS in terms of *IGHV@* and *IGHJ@* genes, mutational status, and CDR3. We found that 100% (344/344; 314 predominant and 30 secondary) of rearrangements in *IGHV@* genes were detected equally. In the case of *IGHJ@* genes, 95.64% (329/344) were identical (including differences in alleles). CDR3 amino acid junction sequence was identical in 87.99% (293/333) of rearrangements in those CDR3s characterized using SSeq. We inspected 17 rearrangements whose sequences were discordant between NGS and SSeq in a single amino acid, querying both consensus sequences through IMGT/V-QUEST. In all of them, SSeq data exhibited an incomplete JH 3′ region and/or failed to identify tryptophan 118 (Trp118), whereas the NGS counterpart consensus sequences given as output by the pipeline for these specific rearrangements showed complete in JH and unambiguous Trp118 identification (further confirmed by IMGT/V-QUEST analysis) (Table S4).

### Mutational status

Mutational status (UM or MM) classification was equal in 99.1% (341/344) of the rearrangements. Three rearrangements differed in mutational status between SSeq and NGS due to a mutation before the FR1 region that could not be detected with the primers employed in the NGS protocol. Their statuses varied from borderline (97.92%, 97.59%, and 97.57% identity) to UM (98% identity).

### Complementary DNA library performance

A limitation of this study was the impossibility to include RNA material for performing the validation. However, since the use of leader primers is advised as per the clinical guidelines [6], we have optimized a variation of the protocol for RNA including leader primers + FR primers (Figure S19).

The in-house pipeline was used to analyze the data with an additional amplicon covering the region from leader to FR1. All cases were determined equal in mutational status, *IGHV*–*IGHJ* alleles, and CDR3 compared to SSeq using this alternative protocol with 6 CLL samples (**Table 2**, Table S5).

**Table 2:** Summary of B-cell clonal rearrangements using an alternative cDNA protocol in six CLL samples

Sample	HTS	No. of leader-mapped reads	No. of FR1-mapped reads	No. of FR2-mapped reads	No. of FR3-mapped reads	Clonal percentage (%)	MS	CDR3	*N*CLONE
cDNA1	IGHV1-2*04_IGHJ6*02	8757	2877	4110	18,365	98.8	UM	CARDGYDILTGYPQDYYYYYGMDVW	1CLONE
cDNA2	IGHV1-69*09_IGHJ4*02	10,079	2224	5326	21,120	98.5	UM	CARAYYDFWSGYSEFDYW	1CLONE
cDNA3	IGHV5-10-1*03_IGHJ4*02	21,112	2383	7604	28,633	100	MM	CARHWGRAWNYRPDYW	1CLONE
cDNA4	IGHV1-69D*01_IGHJ6*02	2021	392	803	3907	98.8	UM	CARSPYCSSTSCYLVDYYYGMDVW	1CLONE
cDNA5	IGHV3-7*04_IGHJ6*02	17,013	4430	14,679	39,756	95.9	MM	CARALSEGYCPSCGMDVW	1CLONE
cDNA6	IGHV3-11*06_IGHJ5*02	15,810	2100	9262	36,415	99.9	UM	CAREKLIYYGSGSYYNWFDPW	1CLONE

*Note*: cDNA, complementary DNA; CLL, chronic lymphocytic leukemia; HTS, high-throughput sequence; FR, framework region; CDR3, complementary determining region 3; MS, mutational state; UM, unmutated; MM, mutated.

## Discussion

Approximately 10% of CLL patients are reported to present more than a single dominant clone, whose relative abundance is not quantifiable using the standard SSeq method [23]. Plevova et al. described at the single-cell level that cases of CLL patients with double productive rearrangements arise from independent B-cell clones, and their outcome was similar to UM CLL patients (earlier need for treatment) [24]. For this reason, in the European Research Initiative on CLL (ERIC) updated guidelines [6], the use of NGS to characterize the relative abundance after the presence of multiple productive rearrangements is advised.

In NGS, the division line between second predominant or multiple rearrangements and subclones remains to be established. In other studies, a fixed clonal percentage after B-cell polyclonal background calculation has been applied [9], whereas in our method, the reliable cut-off defined after the test dataset depends on the clonality profile of each sample. To minimize FN and FP clones, the clonal threshold required fine-tuning according to the abundance of the clones within a sample. The results show only FPs and no FNs.

Detection of multiple rearrangements by SSeq is more tedious and less straightforward, as each *IGHV@* family must be amplified individually, using different primers and sequenced separately. Herein, there are two samples with multiple productive rearrangements that were not detected primarily by SSeq (only after NGS result validation) (Table S1), and in the additionally confirmed clone n.8 (Figure S9), SSeq quality was poor and therefore the rearrangement could only be characterized using NGS.

Regardless of the productivity, cases with multiple rearrangements from the same *IGHV@* family are a technical limitation undetected by SSeq if DNA fragments are close in sequence length, therefore obtaining noise from various clones after sequencing. In 88.9% (8/9) of cases, the additional rearrangements detected using only NGS are due to this limitation.

In 2.5% (5/9) of the rearrangements classified as FP after GeneScan clonality analysis, the artifact clones shared CDR3 amino acid sequence with the major confirmed rearrangements. These were not detected with MiXCR and therefore, there is a confirmation that they are caused by unspecific *IGHV@* gene mapping that were not solved after grouping high-similarity rearrangements. Therefore, they should not be considered as different clones in practice and can be spotted after inspection. MiXCR is advantageous in minimizing the FPs. However, it does not provide a delineation of the clonally expanded rearrangements and therefore the FP detection was judged by comparing the *IGHV@* genes of the rearrangements reported by B-MyRepCLL, and not in a systematic manner. On the other hand, MiXCR does not report allele detail on *IGHV* assignment, and therefore would make the determination of SHM less straightforward.

As it has been described, subclonal rearrangements can be stable over time or can become dominant due to clonal competition, especially after treatment [25]. Characterizing the subclonal architecture is gaining importance, as studies using BCR NGS and single-cell analysis have frequently revealed the presence of clonal heterogeneity, an interesting area of study, including cases of intraclonal diversity evidenced by the presence of a dominant rearrangement surrounded by minor “satellite” clones [13,26,27]. The subclonal fraction remains available for inspection with our tool. However, to quantify the degree of SHM in subclones, higher coverage sequencing and adding unique molecular identifiers (UMIs) will be required.

Amplification biases and mispriming can occur with the multiplex polymerase chain reaction (PCR) approach for sequencing immune repertoires. *IGHV@* standard primers hybridize only partially with many alleles and favor higher rates of amplification in others [8]. Using the three FR primer sets in a single multiplex reaction ensures that rearrangement and the clonality characterization are detected whenever possible. Although guidelines recommend using leader primers for covering *IGHV@* region entirely [6,8], other studies have previously described no significant difference using the same approach with FR1 primers [12,28]; in our case, 341 of the 344 rearrangements (99.1%) were concordant with the primer sets employed, and other 3 samples (0.9%) were classified as UM (98% identity), whereas they were determined as borderline by SSeq.

Commercial kits are available for detection of B-cell clonal rearrangements, such as the LymphoTrack Assay (Invivoscribe, San Diego, CA). The kits available for *IGHV@* mutational status determination are *IGH* leader and *IGH* FR1, which require the use of 2 × 300 and 2 × 250 sequencing kits, respectively. Using 2 × 150 bp improves analytical turnaround time (> 24-h reduction) and NGS data quality, which is highly beneficial for clinical decision making. In addition, sequencing can be combined with other clinical gene panels in the same sequencing experiment [21], and be scaled into Illumina machines with higher capacity.

BCR sequencing requires high read quality due to its inner variability, and differences between real SHM and sequencing artifacts are difficult to assess [29]. The consensus sequence strategy used in the analysis pipeline described permits the correction of sequencing and PCR errors after the reconstruction of the VDJ sequences from the three FR amplicons (Figure S21).

Over the years, NGS is becoming more present in CLL clinical determinations, as reflected in the latest ERIC guidelines update, although a complete protocol has not been standardized [6]. The method presented herein has been applied for sequencing and analyzing IG clonal rearrangements in CLL, reaching high sensibility and specificity. We used our own analysis pipeline specifically developed for the analysis of this data, and we made it publicly available. The former allowed us to detect additional rearrangements that were initially under-appreciated with SSeq in a simple, unbiased manner. Moreover, it is a fast, easy procedure reliable for both mutational status and clonality characterization, and we believe that it can be adapted to facilitate its application in clinical laboratories. However, further validation studies using conventional NGS should be performed.

## Materials and methods

### Patients, sample collection and preparation, and DNA/RNA extraction

A total of 314 peripheral blood (PB) and 5 bone marrow aspirate samples from 319 CLL patients were obtained as part of the diagnosis workout. In addition, 47 healthy donor samples from PB were selected as polyclonal control samples. All CLL patients were diagnosed according to the National Cancer Institute Working Group guidelines in our institution between 1986 and 2019. Samples were provided by the INCLIVA Biobank (PT20/00029; B.000768 ISCIII), integrated in the Biobanks and Biomodels ISCIII Platform and in the Valencian Biobanking Network and they were processed following standard operating procedures with the appropriate approval of the Ethics and Scientific Committees. The gDNA was isolated by the Promega Maxwell 16 Blood DNA Purification Kit (Catalog No. AS1290, Promega, Madison, WI).

### Classical PCR SSeq method

For SSeq assessment of *IGHV@* mutational status, gDNA (50–100 ng) was amplified using *IGH* locus-specific primer sets (leader or FR1 primers and consensus JH primers), as described in the guidelines [30,31]. The presence of rearranged bands was checked by means of capillary electrophoresis by QIAxcel Advanced system (Catalog No. 9001941, QIAGEN, Hilden, German). Direct sequencing of the PCR reaction with forward and reverse primers was advisable. SSeq was performed with BigDye Primer Sequencing Kit (Catalog No. 4337455, Thermo Fisher Scientific, Waltham, MA). The IMGT/V-QUEST tool [32] was employed for the analysis method, following clinical guidelines [6].

### Multiplex PCR NGS methods

#### DNA

DNA (50 ng) was amplified using a mix of primer sets in multiplex to obtain nested fragments in a single reaction. This reaction includes the previously described sets of FR1, FR2, and FR3 primers (Figure 1A).

A second PCR was performed with adapter sequences. Samples were pooled, purified using Magsi-NGS Prep magnetic beads (Catalog No. MD-61021, Magnamedics, Geleen, Netherlands), and sequenced using Illumina MiSeq v2 150 × 2 sequencing kit (Catalog No. MS-102-2002, Illumina), following manufacturer’s specifications.

#### RNA

A second mix of oligonucleotides was prepared by adding the leader primer set to the mix described above. Leader primers ensure complete coverage of the *IGHV@* region, but intron 1 present downstream the leader region does not allow to sequence the rearranged complex from gDNA using 150 bp reads as it does not reach *IGHV@* exon 2. For that reason, we included the set of leader primers set for an alternative approach from complementary DNA. Six CLL samples were amplified with the primer mix and sequenced as described above.

### Capillary fragment analysis

The NGS results identified B-cell rearrangements not previously detected using the standard SSeq protocol. In seven cases where the additional and previously-detected rearrangements belonged to the same *IGHV@* family, SSeq was repeated as previously described. Afterward, in cases where there were still incongruences, along with cases of coexisting multiple rearrangements from the same family, amplification with leader, FR1, FR2, or FR3 consensus primers followed by fragment length analysis (GeneScan) was performed on ABI3730 capillary DNA analyzer (Applied Biosystems, Waltham, MA)

### Bioinformatics analysis

#### Pipeline basis

An in-house pipeline was developed to detect clonal B-cell rearrangements from NGS data, using the reference IMGT (the international ImMunoGeneTics information system) VDJ allele database [33]. The high clonal representation of one or a few rearrangements defined by the nature of CLL pathobiology motivated the use of a consensus sequence strategy (Figure 2), tailored to the NGS libraries designed herein.

Analyses were performed using an in-house pipeline (https://github.com/afuentri/B-MyRepCLL). To overcome partial sequencing with 150 read length, sequences from three different amplicons were integrated at various steps of the workflow to characterize the VDJ region from the *IGH* locus. Also, fragment-wise information (distinguishing reads between FR1, FR2, or FR3 fragments) was used to ensure complete sequence information.

#### Rearrangement detection and characterization

The main steps of the pipeline consisted of mapping reads simultaneously against the *IGHV@* and *IGHJ@* IMGT allele database after the preprocessing steps (trimming ends below Q30 of Phred score and minimum read length 50 bp) (Figure 2). The alleles represented in each sample were kept in comma-separated values (CSV)-formatted files as read counts. Read support for each FR amplicon was counted to detect sequencing artifacts and fragment biases. The next step was to define complete-length IG rearrangement information by extracting read mapping information simultaneously against *IGHV@* and *IGHJ@* alleles. After determining *IGHV@*–*IGHJ@* correspondence, reads were isolated to obtain a consensus sequence per *IGHV@*–*IGHJ@* rearrangement (in this case approached with paired *IGHV@*–*IGHJ@* allele information) found in one sample. For this purpose, reads belonging to either of the paired *IGHV@* and *IGHJ@* alleles were mapped against a simulated rearrangement reference made of concatenated *IGHV@*–*IGHJ@* germline allele sequences. Rearrangement consensus sequences were aligned (pairwise local alignment) against germline IMGT *IGHV@* alleles to annotate the percentage of identity and the mutations found. Alignment against a rearrangement-specific sequence (pairs of *IGHV@*–*IGHJ@* alleles) was used to delimit the *IGHD@* nucleotide sequence, inferred as an insertion. CDR3 sequences were extracted from the main unique sequence by seeking conserved amino acid motifs (Cys104, Trp118, and WGXG).

#### Artifact filtering

To avoid artifacts deriving from nonspecific *IGHV@* gene mapping, first alleles and secondly genes whose consensus sequences shared ≥ 95% identity were joined into the predominant IG rearrangement. Afterward, *IGHV@* alleles supported by only one fragment or by FR3 amplicon with at least 92% of total reads were added to the major allele with equal *IGHV@* family and CDR3. Rearrangement information was summarized at the gene level of *IGHV@* in the final report while keeping the detail at the allele level (Figure S20).

### Filtering and interpretation

Three hundred and nineteen samples with > 1000 total reads assigned to the major IG rearrangement were selected for validation against the gold standard method. Five samples were unavailable for validation at the capillary fragment analysis stage and were removed from the study. Polyclonal samples were selected with a minimum of 1000 total reads after trimming (47 samples).

### Reliable cut-off test

After detecting and accordingly characterizing predominant pathological clones, reaching a reliable cut-off for NGS minor clones was necessary to report only CLL clonal IG rearrangements and differentiate the rest as subclonal background. For this purpose, we trained a KNN machine learning model employing Python scikit-learn KNeighborsClassifier to classify healthy and clonal profiles. The maximum difference read ratio between consecutive clones (MAX_DIFF parameter) in samples with clonal profiles was used to adjust the cut-off for these test samples. The following formula was applied: %reads_mapped (*N*)/%reads_mapped (*N* + 1) (*N* being the current clone and *N* + 1 being the next consecutive clone in abundance order). The maximum of these ratios was the clonal cut-off (MAX_DIFF parameter) (Figure 1C).

Mann-Whitney U test was used to determine significant differences between the maximum difference ratios obtained in pair comparisons between the three groups. After this classification, samples are tagged as “polyclonal” or “*N*CLONE” (with *N* representing the number of potential pathological CLL clones). Clones with a percentage < 0.1% were filtered out prior to classification for their low proportion to reduce noise. Inconsistencies with SSeq were assessed and additional IG rearrangements detected by NGS were validated using fragment capillary sequencing as described in the “Capillary fragment analysis” section. Rearrangements confirmed both by NGS and SSeq were subjected to comparison regarding *IGHV@*, *IGHJ@* genes, mutational status, and CDR3 sequence.

To validate the BCR rearrangement information of the clones that were not detected by SSeq, we used MiXCR (v4.0) [34]. The module *analyze amplicon* was employed to preprocess and obtain rearrangement information from the FASTQ files and the command *exportClones* was run with parameter “*-vIdentityPercents*” to export into delimited table format with the V alignment identity percentages.

## Ethical statement

This study was approved by the local Hospital Research Ethics Committee. Samples were provided by the INCLIVA Biobank (PT20/00029; B.000768 ISCIII), which is integrated in the ISCIII Biobanks and Biomodels Platform and the Valencian Biobanking Network. Sample processing followed the standard operating procedures with the appropriate approval of the Ethics and Scientific Committees (Approval No. 2021/247).

## Code availability

The workflow employed to fully characterize BCR rearrangements in CLL patients can be accessed on GitHub (https://github.com/afuentri/B-MyRepCLL).

## Supplementary Material

qzaf041_Supplementary_Data

## Data Availability

Data supporting the findings of this study are available from the corresponding author upon reasonable request.

## CRediT author statement

**Azahara Fuentes-Trillo:** Data curation, Formal analysis, Investigation, Methodology, Software, Validation, Visualization, Writing – original draft, Writing – review & editing. **Alicia Serrano-Alcalá:** Formal analysis, Investigation, Resources, Writing – review & editing. **Blanca Ferrer-Lores:** Investigation, Resources, Writing – review & editing. **Laura Ventura-López:** Formal analysis, Investigation, Writing – review & editing. **Enrique Seda:** Investigation, Methodology, Resources, Writing – review & editing. **Ana-Bárbara García-García:** Formal analysis, Investigation, Methodology, Visualization, Writing – original draft, Writing – review & editing. **Blanca Navarro:** Conceptualization, Funding acquisition, Methodology, Project administration, Writing – review & editing. **María José Terol:** Funding acquisition, Methodology, Project administration, Writing – review & editing. **Felipe Javier Chaves:** Conceptualization, Funding acquisition, Methodology, Project administration, Supervision, Validation, Writing – original draft, Writing – review & editing. All authors have read and approved the final manuscript.
