# Supplementary material for: Characterization of Chronic Lymphocytic Leukemia Immunoglobulin Rearrangements from Partial Read Sequencing
Source: Genomics Proteomics Bioinformatics. 2025 May 2;23(2):qzaf041. doi: 10.1093/gpbjnl/qzaf041 (PMC12536063; doi:10.1093/gpbjnl/qzaf041)
Supplement: qzaf041_Supplementary_Data [file qzaf041_supplementary_data.zip › Supplementary material captions.docx]

**Supplementary material**

**Figure S1** **Percentage breadth of coverage above 500 reads in clonal rearrangements (% ranging from 2 to 100)**

Mean coverage breadth 85%.

**Figure S2 Additional clones 1 GeneScan *IGH* clonality analysis**

**Figure S3 Additional clones 2 GeneScan *IGH* clonality analysis**

**Figure S4 Additional clones 3 GeneScan *IGH* clonality analysis**

**Figure S5 Additional clones 4 GeneScan *IGH* clonality analysis**

**Figure S6 Additional clones 5 GeneScan *IGH* clonality analysis**

**Figure S7 Additional clones 6 GeneScan *IGH* clonality analysis**

**Figure S8 Additional clones 7 GeneScan *IGH* clonality analysis**

**Figure S9 Additional clones 8 QIAxcel DNA electrophoresis gel image after amplification of *IGHV3* family with FR1–JH oligonucleotides**

**Figure S10 Additional clones 9 GeneScan *IGH* clonality analysis**

**Figure S11 FP1 GeneScan *IGH* clonality analysis**

**Figure S12 FP2 GeneScan *IGH* clonality analysis**

**Figure S13 FP3 GeneScan *IGH* clonality analysis**

**Figure S14 FP4 GeneScan *IGH* clonality analysis**

**Figure S15 FP5 GeneScan *IGH* clonality analysis**

**Figure S16 FP6 GeneScan *IGH* clonality analysis**

**Figure S17 FP7 GeneScan *IGH* clonality analysis**

**Figure S18 FP8 GeneScan *IGH* clonality analysis**

**Figure S19 Example of IGV VDJ rearrangement BAM file visualization**

**A**. UM case (sample cDNA4 from Table 2), IGHV1-69D*01_IGHJ6*02. **B**. MM case example (sample cDNA3 from Table 2), IGHV5-10-1*03_IGHJ4*02. UM, unmutated; MM, mutated.

**Figure S20** **Rearrangement prioritization and filtering steps performed by B-MyRepCLL**

The first table represents the raw mapping results of reads against *IGHV* alleles differentiated by FR fragment (1), with its corresponding *IGHJ@* and CDR3. From (1) to (2), rearrangements sharing the *IGHV@* gene (but different *IGHV@* allele) and consensus sequence identity ≥ 95% are merged into the same rearrangement, and alleles are kept in the “joined alleles” column. The different alleles from *IGHV3-11* gene are observed to have been joined and the number of reads are added to the major allele *IGHV3-11*01*. From step (2) to (3), the former procedure is repeated at *IGHV@* gene level, assigning the reads of close *IGHV@* genes sharing ≥ 95% identity into the major rearrangement in allele *IGHV3-11*01*. The rearrangements in red are supported mainly by FR3 and share CDR3 and *IGHJ@* with *IGHV3-11*, so reads are assigned to the major balanced rearrangement, showing the major rearrangement highlighted in (4).

**Figure S21 Multiplexed amplification design**

**A.** Multiplex method composed by the combination of the three framework primer sets. **B.** Read distribution obtained for this primer set with 2 × 150 bp Illumina sequencing kit with the 3 FR primer sets.

**Table S1 Samples with multiple rearrangements**

**Table S2 Additional clones confirmed using GeneScan analysis**

**Table S3 Validation false positives (samples with additional artifact rearrangements reported)**

**Table S4 Cases of 17 discordant CDR3 sequences with 1 single amino acid difference after comparison of the rearrangement characterization in the NGS procedure *vs*. SSeq**

**Table S5 B-MyRepCLL output table with clones information in the cDNA test with 6 CLL samples**
