# Supplementary figures and images for: Characterization of Chronic Lymphocytic Leukemia Immunoglobulin Rearrangements from Partial Read Sequencing
Source: Genomics Proteomics Bioinformatics. 2025 May 2;23(2):qzaf041. doi: 10.1093/gpbjnl/qzaf041 (PMC12536063; doi:10.1093/gpbjnl/qzaf041)

**
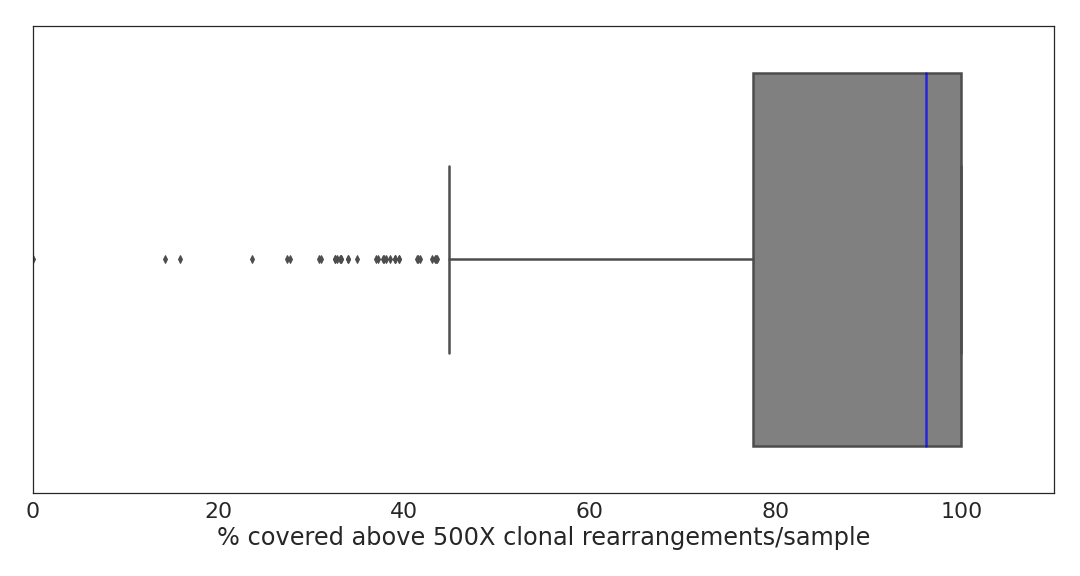
**

Supplement: qzaf041_Supplementary_Data [file qzaf041_supplementary_data.zip › Figure S1.docx]

**
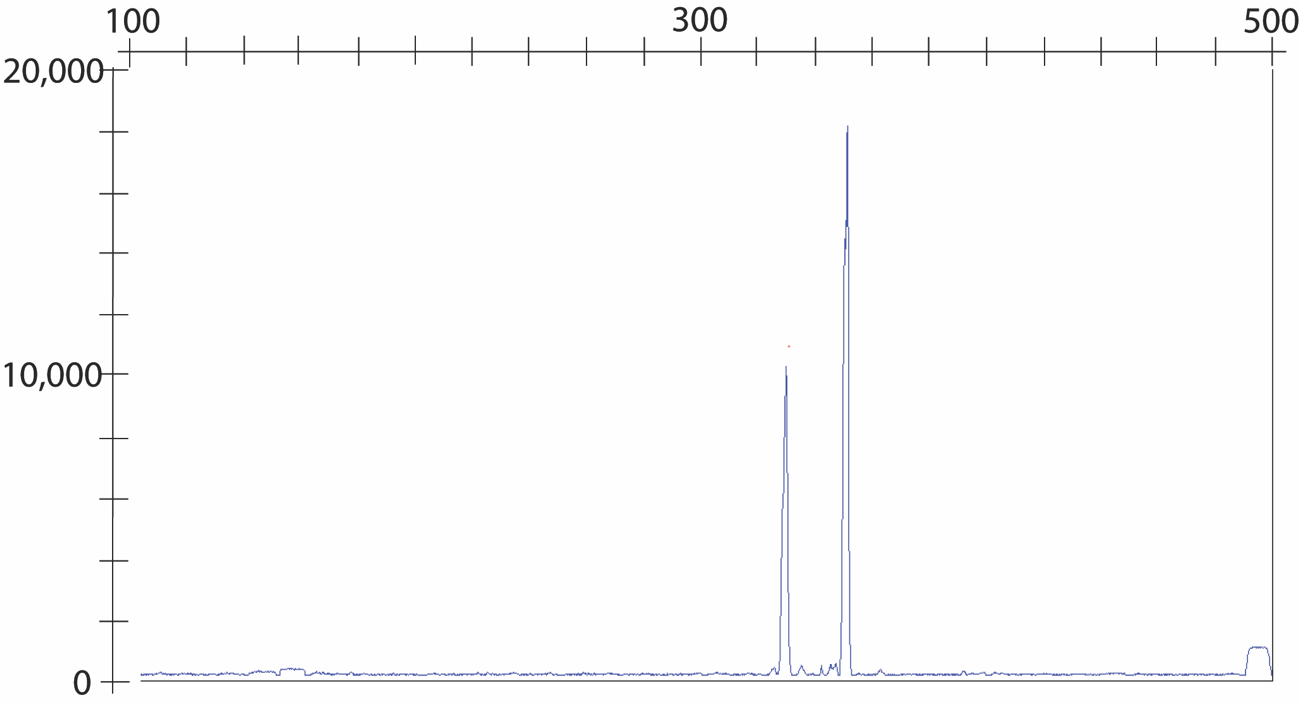
**

Supplement: qzaf041_Supplementary_Data [file qzaf041_supplementary_data.zip › Figure S10.docx]

**
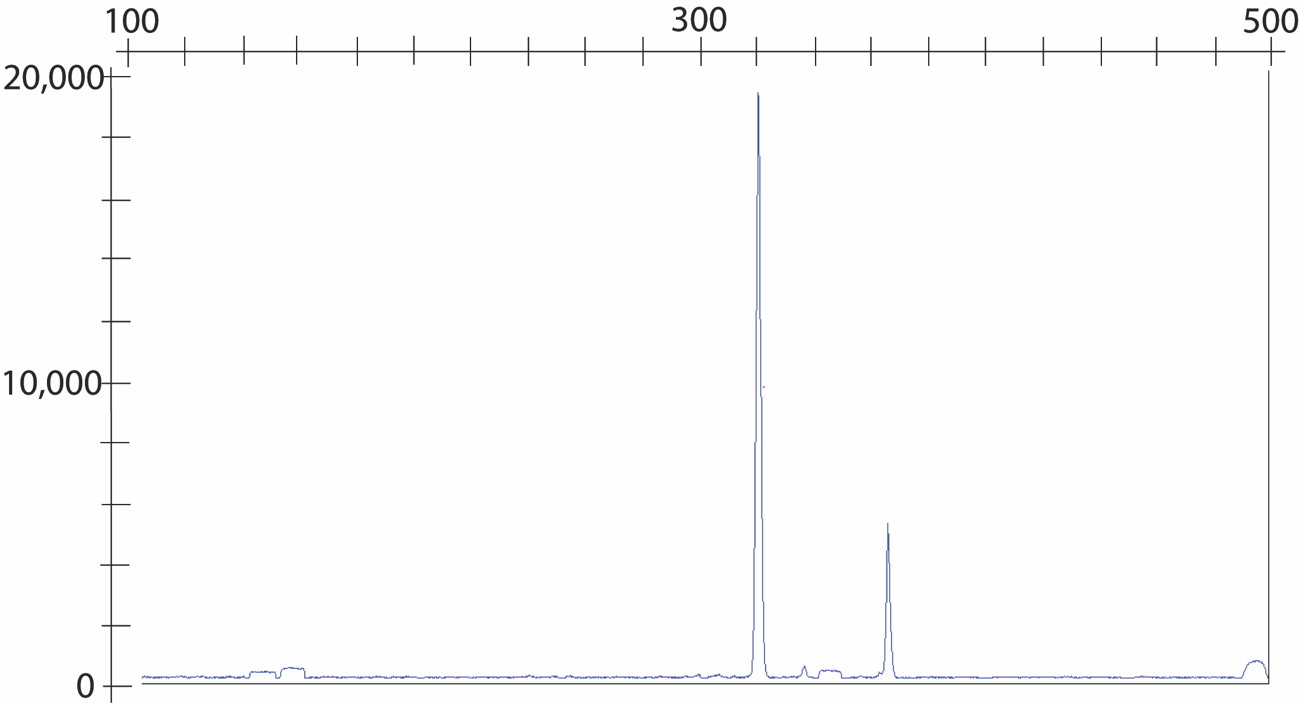
**

Supplement: qzaf041_Supplementary_Data [file qzaf041_supplementary_data.zip › Figure S11.docx]

**
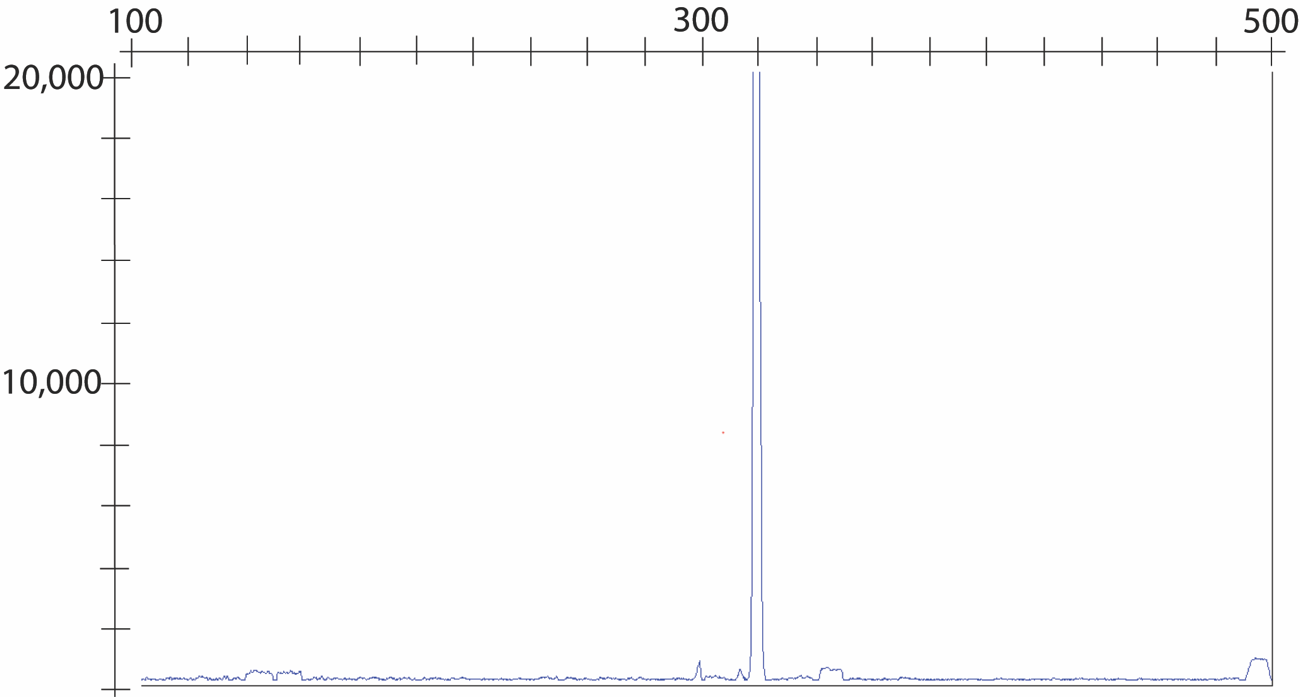
**

Supplement: qzaf041_Supplementary_Data [file qzaf041_supplementary_data.zip › Figure S12.docx]

**
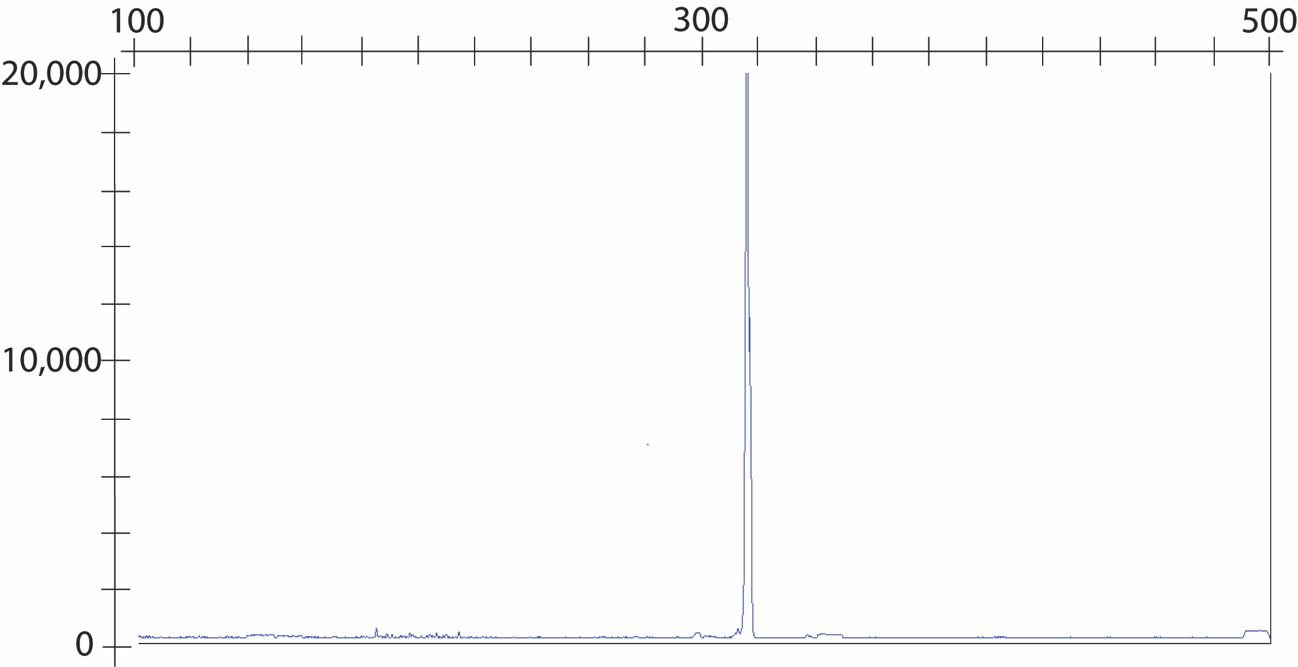
**

Supplement: qzaf041_Supplementary_Data [file qzaf041_supplementary_data.zip › Figure S13.docx]

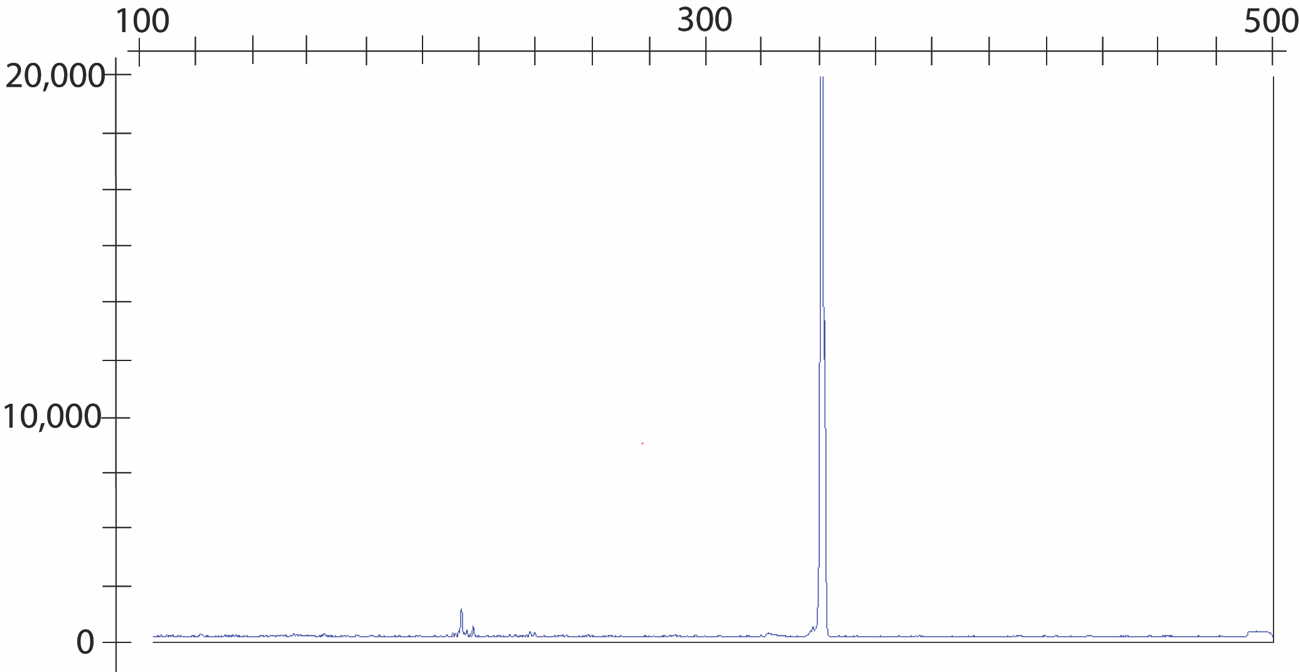

Supplement: qzaf041_Supplementary_Data [file qzaf041_supplementary_data.zip › Figure S14.docx]

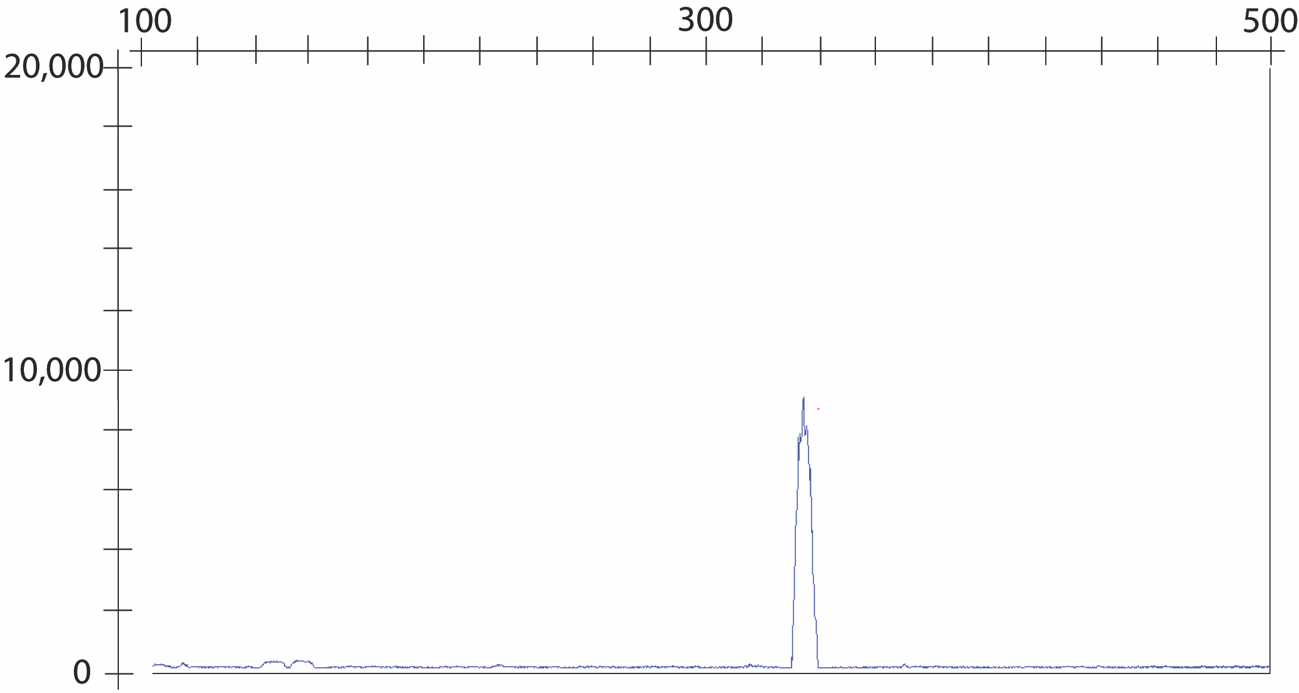

Supplement: qzaf041_Supplementary_Data [file qzaf041_supplementary_data.zip › Figure S15.docx]

500

300

100

6000

3000

0


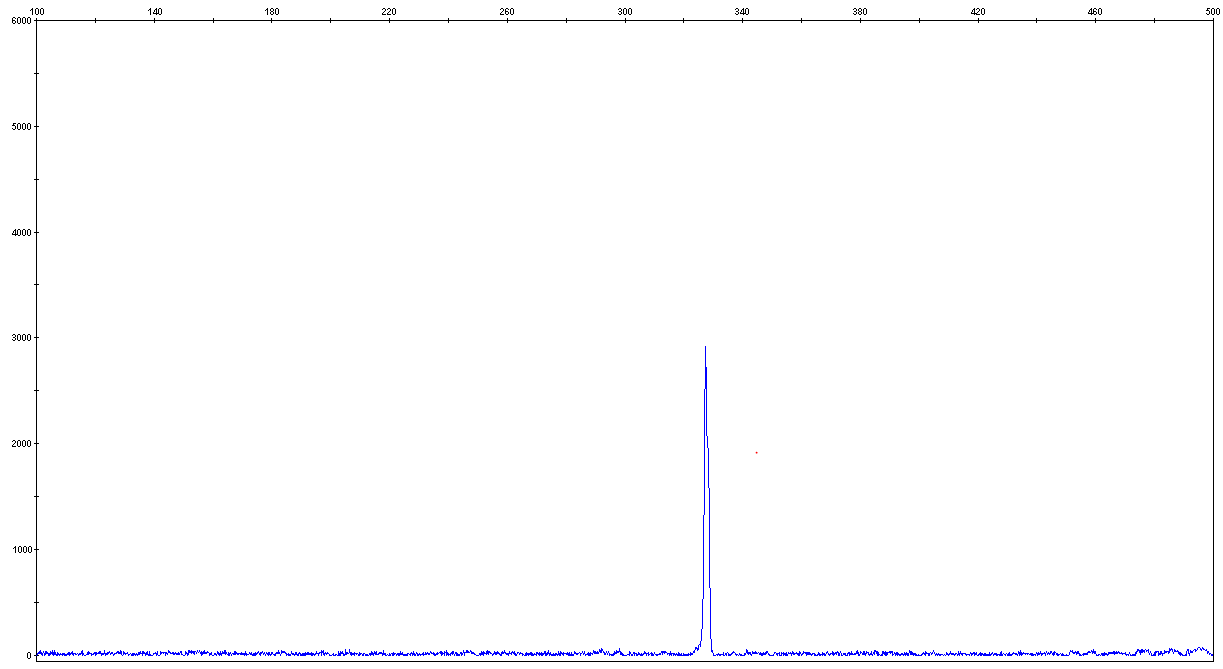

Supplement: qzaf041_Supplementary_Data [file qzaf041_supplementary_data.zip › Figure S16.docx]

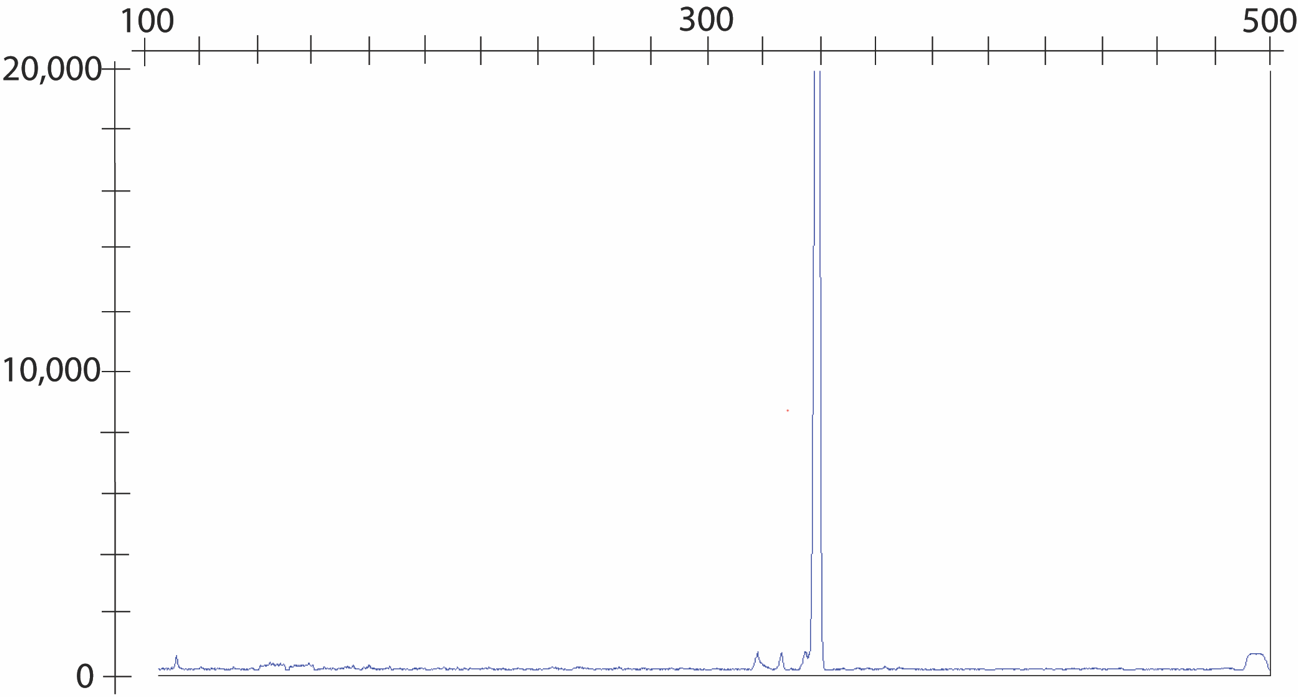

Supplement: qzaf041_Supplementary_Data [file qzaf041_supplementary_data.zip › Figure S17.docx]

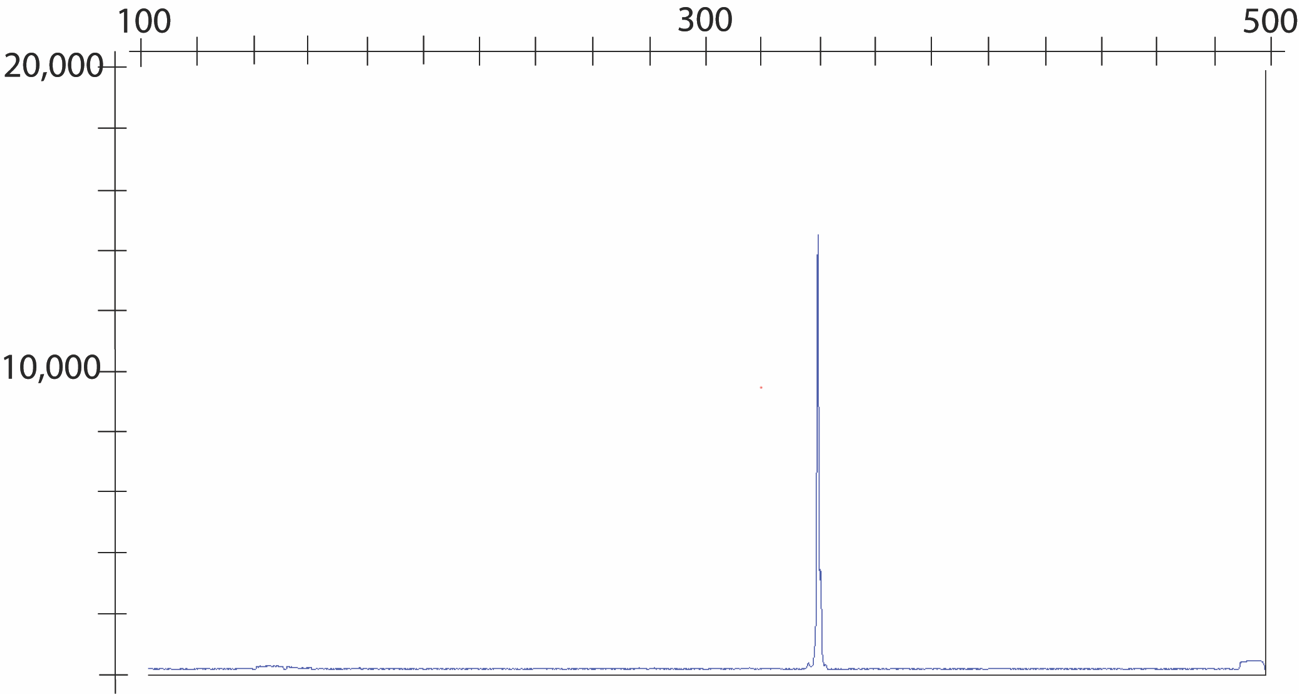

Supplement: qzaf041_Supplementary_Data [file qzaf041_supplementary_data.zip › Figure S18.docx]

**
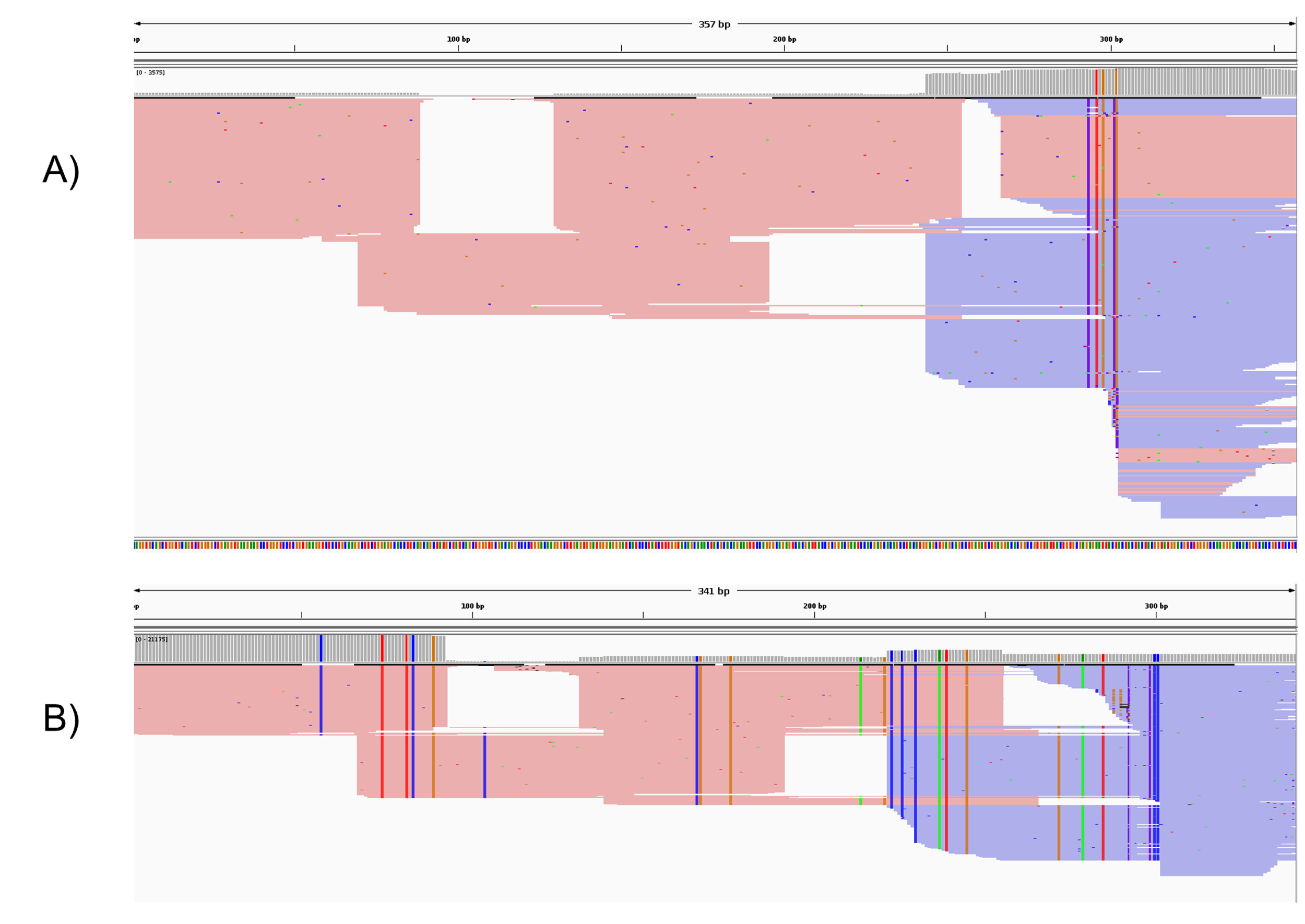
**

Supplement: qzaf041_Supplementary_Data [file qzaf041_supplementary_data.zip › Figure S19.docx]

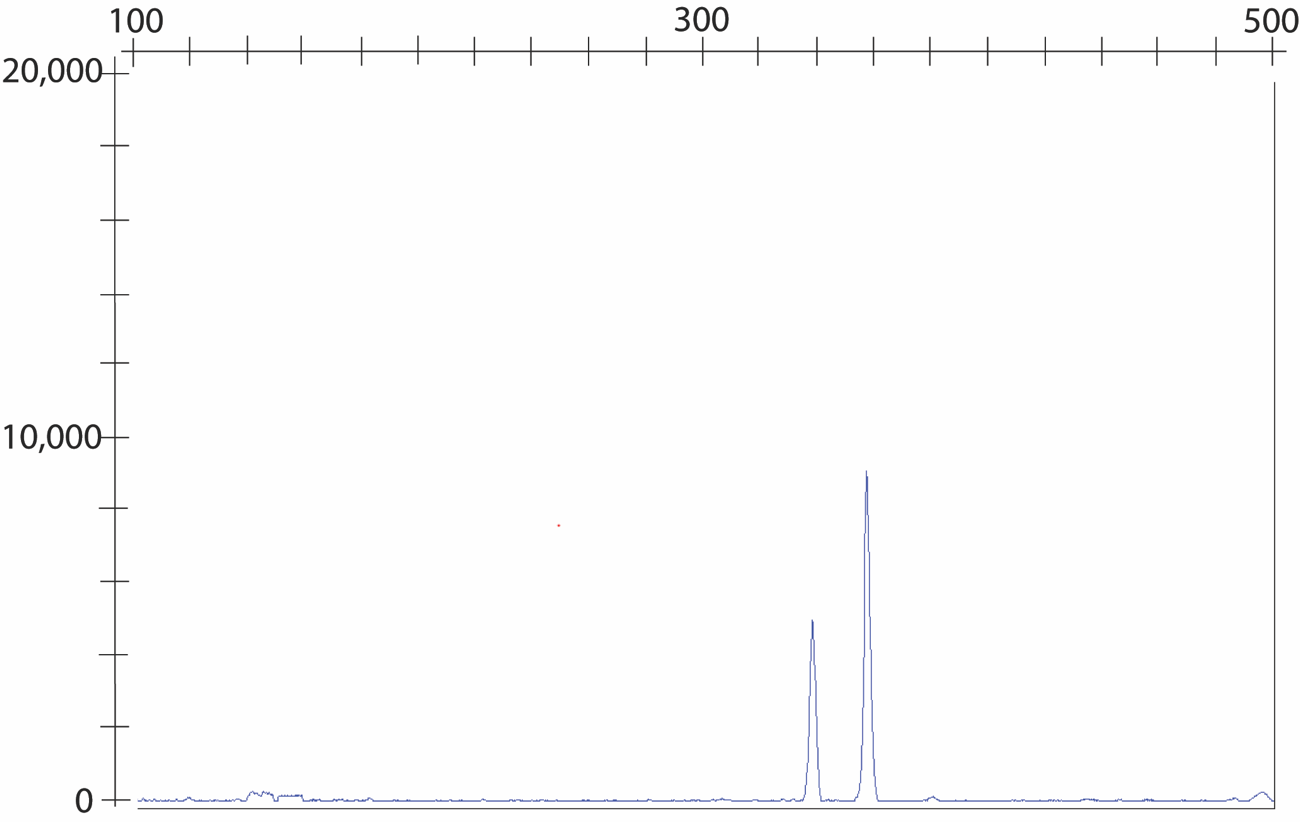

Supplement: qzaf041_Supplementary_Data [file qzaf041_supplementary_data.zip › Figure S2.docx]

**
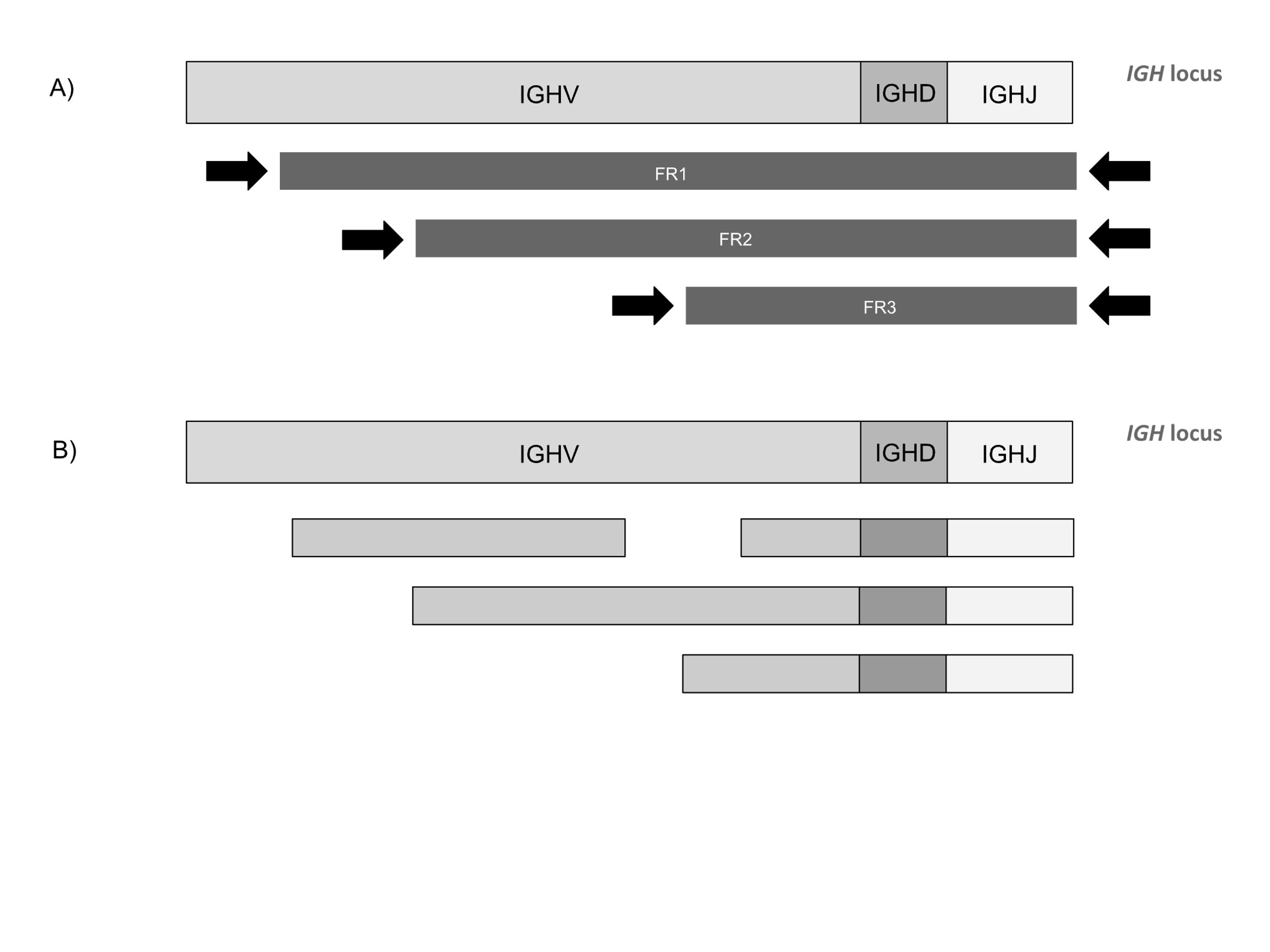
**

Supplement: qzaf041_Supplementary_Data [file qzaf041_supplementary_data.zip › Figure S21.docx]

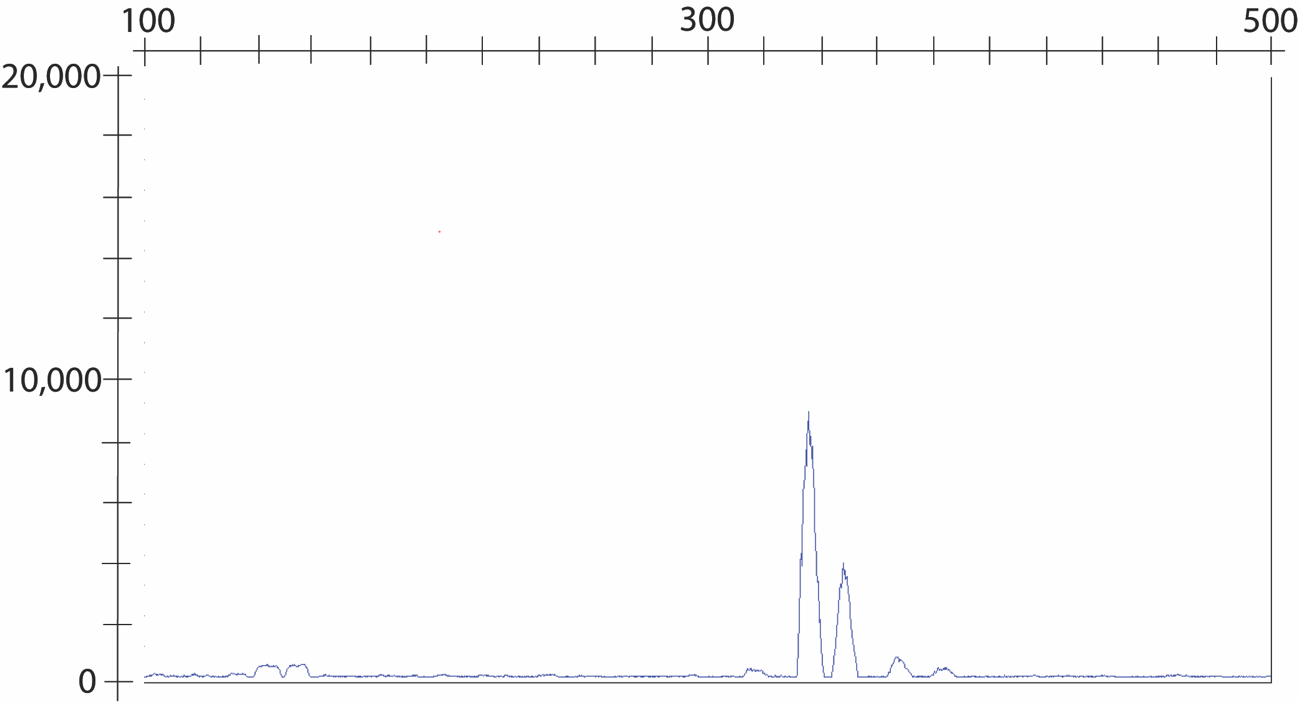

Supplement: qzaf041_Supplementary_Data [file qzaf041_supplementary_data.zip › Figure S3.docx]

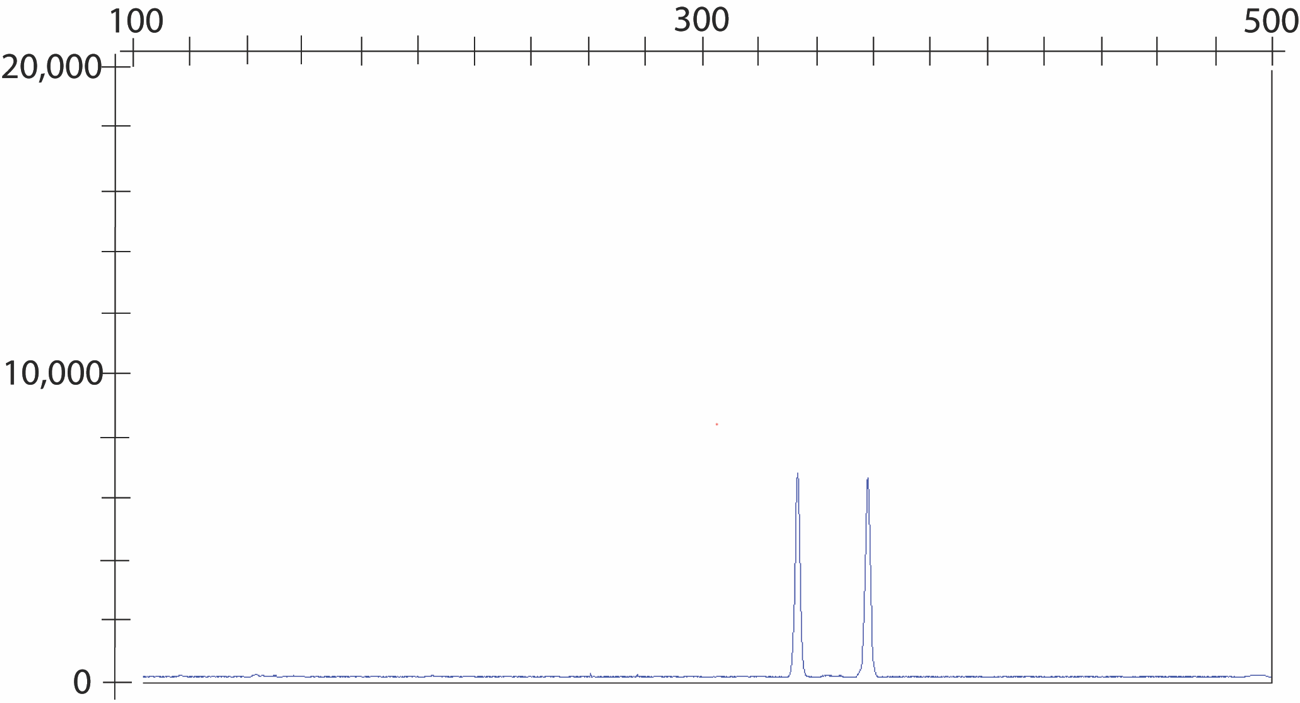

Supplement: qzaf041_Supplementary_Data [file qzaf041_supplementary_data.zip › Figure S4.docx]

500

300

100

1200

600

0


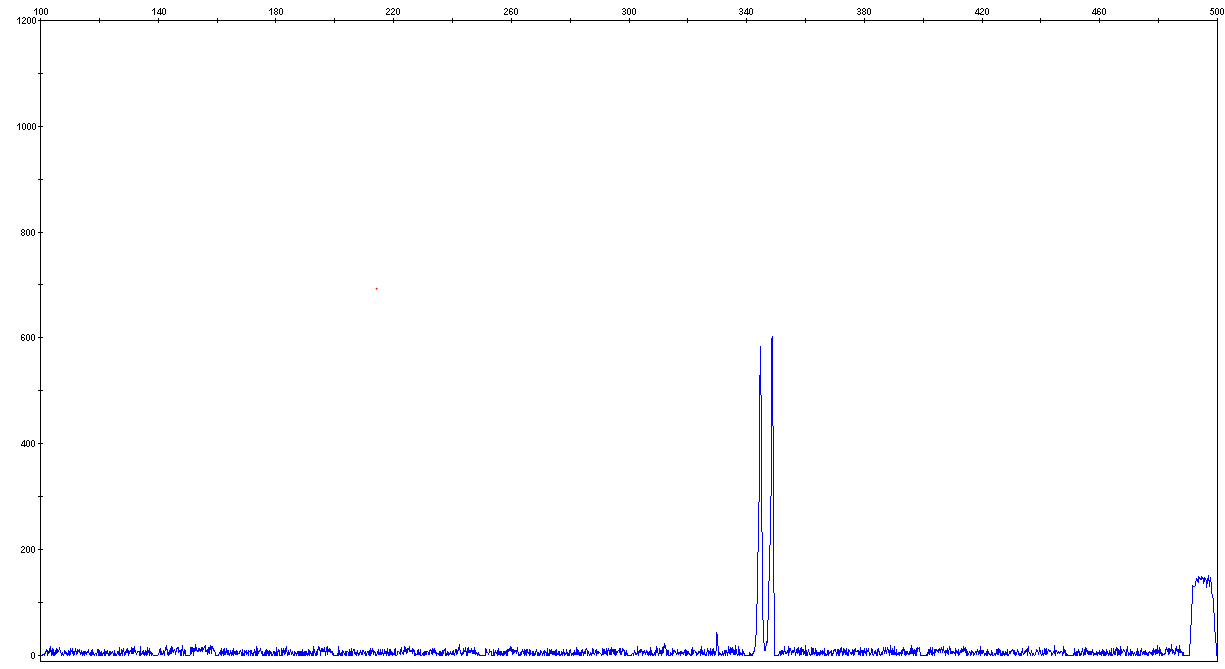

Supplement: qzaf041_Supplementary_Data [file qzaf041_supplementary_data.zip › Figure S5.docx]

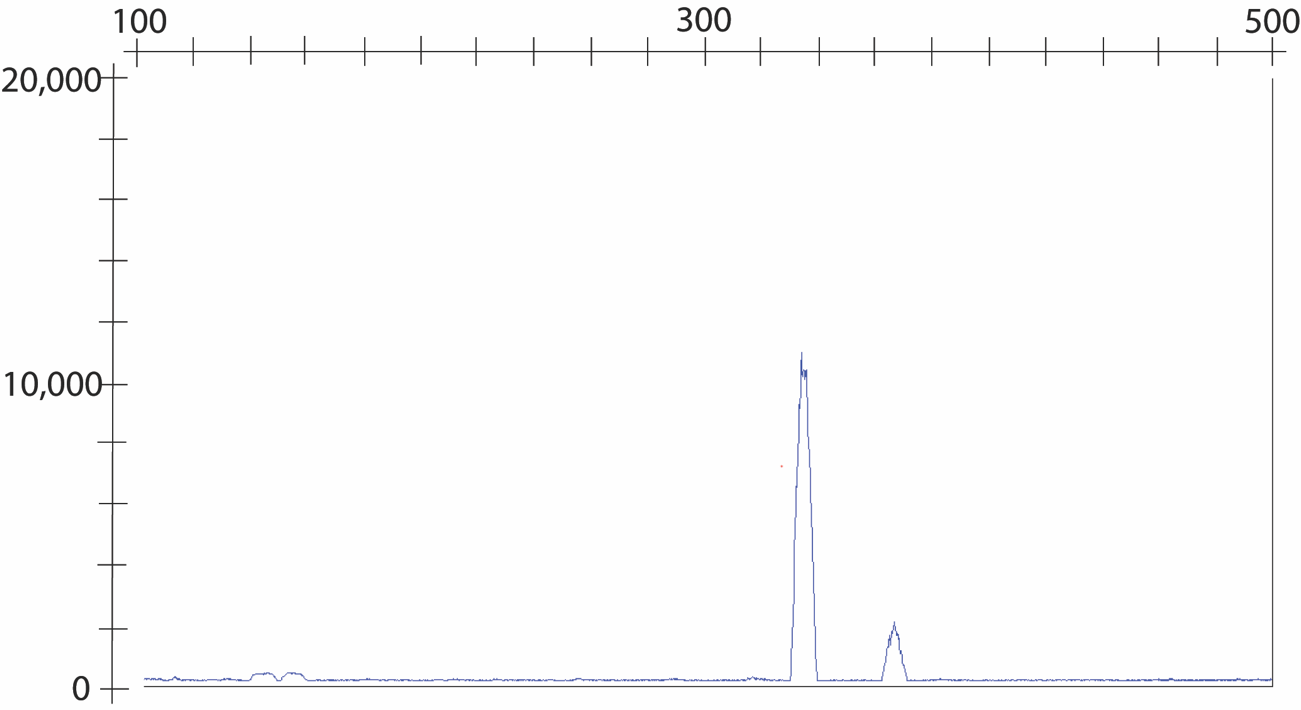

Supplement: qzaf041_Supplementary_Data [file qzaf041_supplementary_data.zip › Figure S6.docx]

500

300

100

8000

4000

0


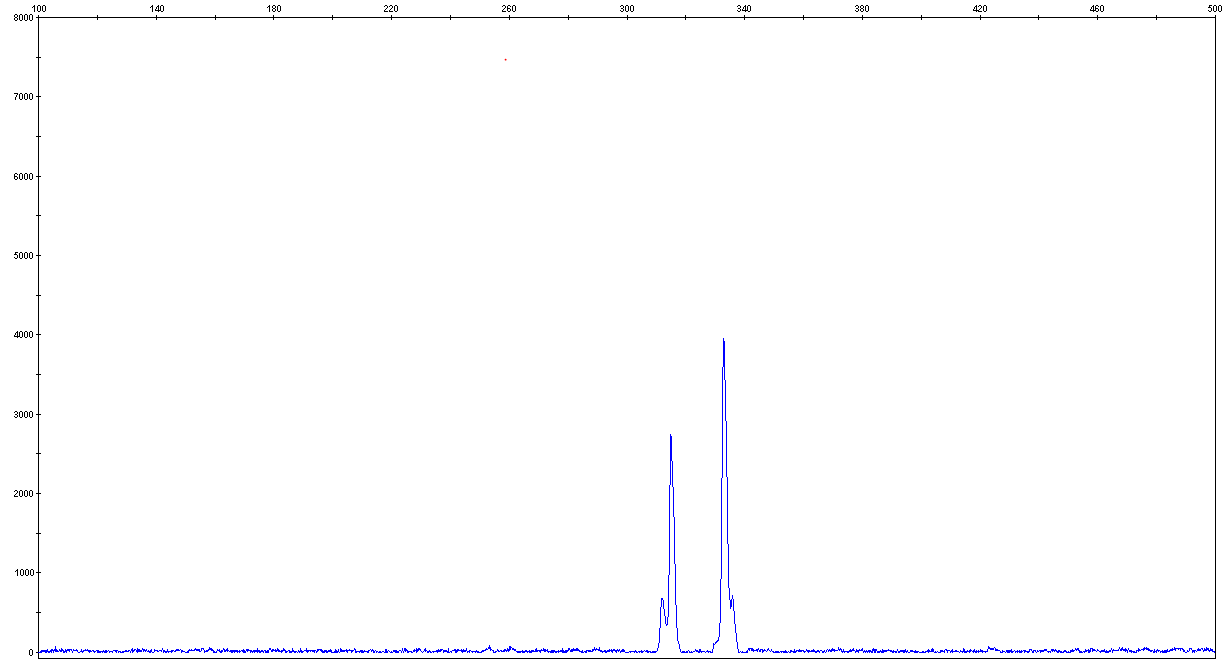

Supplement: qzaf041_Supplementary_Data [file qzaf041_supplementary_data.zip › Figure S7.docx]

500

300

100

8000

4000

0


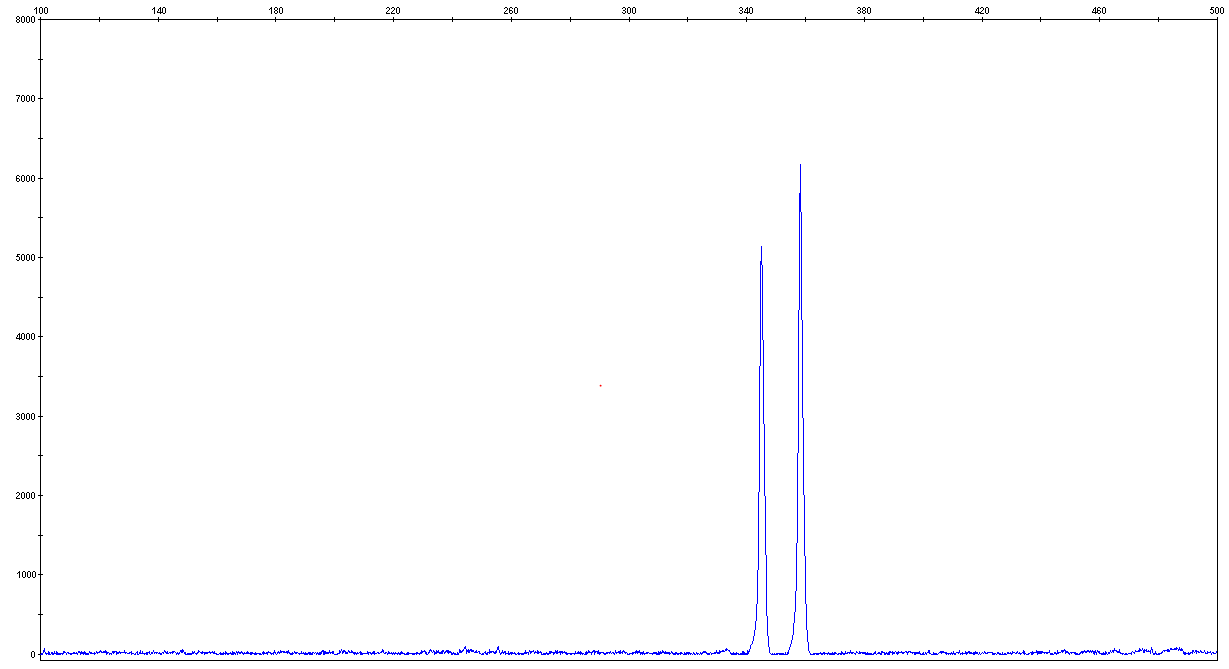

Supplement: qzaf041_Supplementary_Data [file qzaf041_supplementary_data.zip › Figure S8.docx]

| 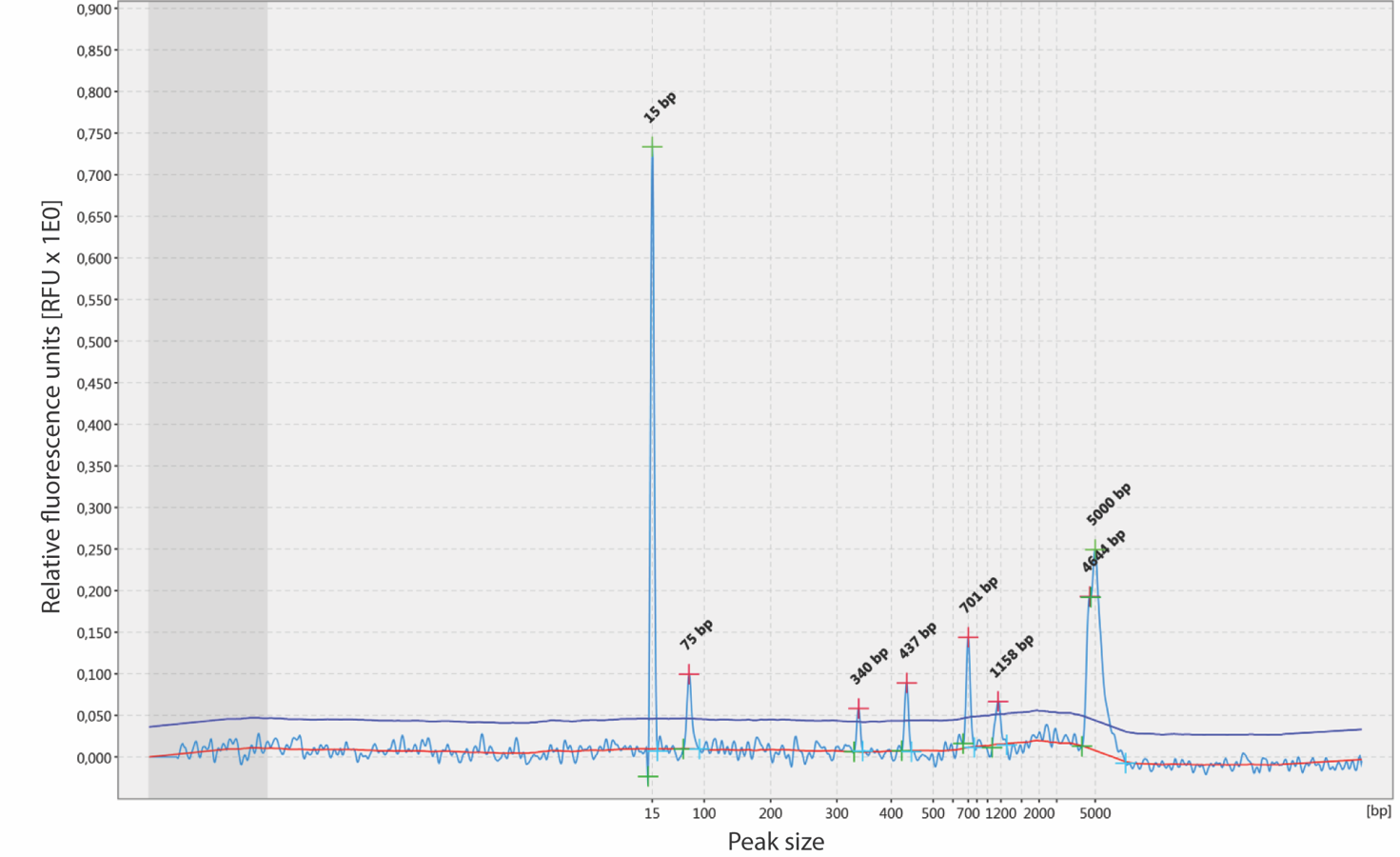 |
| --- |

Supplement: qzaf041_Supplementary_Data [file qzaf041_supplementary_data.zip › Figure S9.docx]
